# Genome-scale identification, classification, and tissue specific expression analysis of late embryogenesis abundant (LEA) genes under abiotic stress conditions in *Sorghum bicolor* L.

**DOI:** 10.1371/journal.pone.0209980

**Published:** 2019-01-16

**Authors:** M. Nagaraju, S. Anil Kumar, Palakolanu Sudhakar Reddy, Anuj Kumar, D. Manohar Rao, P. B. Kavi Kishor

**Affiliations:** 1 Department of Genetics, Osmania University, Hyderabad, India; 2 Department of Biotechnology, Vignan’s Foundation for Science, Technology and Research, Vadlamudi, Guntur, Andhra Pradesh, India; 3 International Crops Research Institute for the Semi-Arid Tropics (ICRISAT), Patancheru, Hyderabad, India; 4 Advance Center for Computational & Applied Biotechnology, Uttarakhand Council for Biotechnology (UCB), Silk Park, Prem Nagar, Dehradun, India; National Institute of Plant Genome Research, INDIA

## Abstract

Late embryogenesis abundant (LEA) proteins, the space fillers or molecular shields, are the hydrophilic protective proteins which play an important role during plant development and abiotic stress. The systematic survey and characterization revealed a total of 68 LEA genes, belonging to 8 families in *Sorghum bicolor*. The LEA-2, a typical hydrophobic family is the most abundant family. All of them are evenly distributed on all 10 chromosomes and chromosomes 1, 2, and 3 appear to be the hot spots. Majority of the *S*. *bicolor* LEA (*SbLEA*) genes are intron less or have fewer introns. A total of 22 paralogous events were observed and majority of them appear to be segmental duplications. Segmental duplication played an important role in *SbLEA-2* family expansion. A total of 12 orthologs were observed with *Arabidopsis* and 13 with *Oryza sativa*. Majority of them are basic in nature, and targeted by chloroplast subcellular localization. Fifteen miRNAs targeted to 25 *SbLEAs* appear to participate in development, as well as in abiotic stress tolerance. Promoter analysis revealed the presence of abiotic stress-responsive DRE, MYB, MYC, and GT1, biotic stress-responsive W-Box, hormone-responsive ABA, ERE, and TGA, and development-responsive SKn *cis*-elements. This reveals that LEA proteins play a vital role during stress tolerance and developmental processes. Using microarray data, 65 *SbLEA* genes were analyzed in different tissues (roots, pith, rind, internode, shoot, and leaf) which show clear tissue specific expression. qRT-PCR analysis of 23 *SbLEA* genes revealed their abundant expression in various tissues like roots, stems and leaves. Higher expression was noticed in stems compared to roots and leaves. Majority of the *SbLEA* family members were up-regulated at least in one tissue under different stress conditions. The *SbLEA3-2* is the regulator, which showed abundant expression under diverse stress conditions. Present study provides new insights into the formation of LEAs in *S*. *bicolor* and to understand their role in developmental processes under stress conditions, which may be a valuable source for future research.

## Introduction

Environmental stresses such as drought, salinity, high and low temperatures, metals, radiation, and diseases cause extensive damage to crop plants by bringing about the changes in gene regulation and metabolism leading to reduced productivity [1]. To combat the stress conditions, plants develop defense-responsive pathways with the help of regulatory and functional genes [2]. Among them, the functional group of genes, mostly the Ca^2+^-dependent signaling molecules activate the late embryogenesis abundant (*LEA*)-type genes, which are protective proteins that help in damage repair of plants under diverse abiotic stress conditions [3]. LEA proteins are characterized by repeated motifs and disordered structure [4], first discovered in cotton seeds [5] during embryo development. Under desiccation, expressions of *LEAs* were high in embryos during seed maturation [6–7]. *LEAs* have been reported to be responsive to various developmental processes and also to abiotic stresses like drought, low temperature, salt, and ABA [8–9]. *LEAs* act as membrane protectors and stabilizers, ion chelators, hydration buffers and antioxidants [9].

Based on their amino acid sequence, homology, and conserved motifs, LEA proteins are classified into eight groups LEA-1, LEA-2, LEA-3, LEA-4, LEA-5, LEA-6, dehydrins, and seed maturation proteins (SMP) [10–11]. The groups 1–5 represent the major groups, and are present in most of the plants [12]. The LEA-1 proteins contain a 20-amino-acid motif (GGETRKEQLGEEGYREMGRK) with a high content of Gly, Glu, and Gln residues [13]. The group 2 dehydrins consist of a motif called K-segment (EKKGIMDKIKEKLPG), which gives chaperone activity to protect proteins during abiotic stress [6]. The LEA-3 proteins have 11-amino acid sequences (TAQAAKEKAGE) repeated 13-times and most of their functions were studied in transgenics [12]. The LEA-4 group does not show any conserved motif or repeats but have a conserved structure at the N-terminus which forms α-helical structure [7]. The LEA-5 has lesser amino acid homology, but participates in seed maturation and dehydration [6]. The LEA-6 proteins are characterized by their small size and two highly conserved motifs, motif 1 with LEDYK but replaced by proline and threonine at positions 6 and 7 in motif 2. Members of dehydrins are intrinsically unstructured, expressed during the late embryogenesis stage and stable under heat stress [14]. LEA proteins are ubiquitous and localized in cytoplasm, nucleus, chloroplast, mitochondria, and endoplasmic reticulum [15]. LEAs do not have a specific localization and their particular functions depend on their intra-cellular locations. For example, the mitochondrial localized pea LEA3 proteins protect rhodanese and fumarase from inactivation under dehydration [16]. On the other hand, the nuclear localized LEA2, LEA4, and LEA7 proteins display DNA binding [17]. The histidine-containing motifs in LEA2 and LEA4 proteins are responsible for binding divalent cations and ion sequestration [18]. LEA proteins are rich in glycine, glutamate, lysine and threonine but lack cysteine and tryptophan residues. Due to the presence of highly charged amino acids like alanine, serine/threonine, they are highly hydrophilic in nature. The secondary structures in LEA proteins are detected by the presence of repeated motifs [9]. Though highly disordered, they acquire structural folding into α-helical conformations under desiccation [19]. Based on the sequence similarity and conserved motif sites, 51 LEA genes belonging to 9 different groups were identified so far in *Arabidopsis* [6–7], 108 in *Brassica napus* [20], 53 in *Populus* [21], 36 in *Brachypodium distachyon* [22], 34 in *Oryza sativa* [23], 30 in *Prunus mume* [24], 29 in *Solanum tuberosum* [25], and 27 in tomato [26].

Over expression of *LEAs* confer abiotic stress tolerance in different plants like *Arabidopsis*, tobacco, rice, wheat, and lettuce [27]. The *NtLEA7-3* shows resistance to drought, salt, and cold in *Arabidopsis thaliana* [28]. In yeast, tomato *LEA25* enhances the salt and chilling stress tolerance [29]. The HVA1 promotes drought and salt stress tolerance in wheat and rice [30–31]. Heterologous expression of *BnLEA4-1* in *E*. *coli* shows tolerance to heat and salt stress [32]. The citrus dehydrin acts as radical scavenger and reduces the metal toxicity [18]. Likewise, two soybean LEA4 proteins bind to Fe and are associated strongly in reducing oxidative damage induced by abiotic stress [33]. Further, it was shown that loss of LEA4 proteins result in drought susceptibility in *Arabidopsis* [34]. The *Arabidopsis LEA2* protein alters the pathogenesis-related protein expression and confers defense response [35]. Similarly, the group 3 LEA proteins in maize confer tolerance to bacterial infection. While their heterologous expression in tobacco exhibit tolerance to *Pseudomonas syringae* [36], wheat *TaLEA2* and *TaLEA3* in yeast enhance the salt and freezing stress tolerance [37]. Lin et al [38] found that *VrDhn1* stabilizes the DNA under seed desiccation. Thus, it appears overexpression of diverse *LEA* proteins offer tailored protection against abiotic stress in a wide range of plants [15].

*Sorghum bicolor* is the fifth most important cereal crop, used as food, feed, fuel, fibre, and fertilizer. It is moderately tolerant to drought, salinity, water logging conditions as well as high temperature [39–42]. The knowledge about the number of LEA proteins and their families, structure characterization, tissue specific expression, and chromosomal location is meagre in *S*. *bicolor*. Hence, in the present investigation, comprehensive genome-scale identification of *LEAs*, their structural characterization, chromosomal location, and promoter analysis alongside the tissue specific gene expressions were carried out under varied abiotic stress conditions.

## Material and methods

### Identification, chromosomal localization, and gene structure analysis of *LEA* in *S*. *bicolor*

In the present study, 34 *Oryza* [23] and 51 *Arabidopsis* [7] *LEA* gene sequences were retrieved from NCBI database and searched (using TBLASTN) against *Sorghum bicolor* genome in Gramene database (http://www.gramene.org/) to find out their homologs. Genscan (http://genes.mit.edu/GENSCAN.html) program was used to retrieve the coding and protein sequences. Based on homology, *Sorghum* LEA sequences were analyzed by SMART program (http://smart.embl-heidelberg.de/) [43] for the presence of conserved domains. MOTIF search (http://www.genome.jp/tools/motif/) tools were used to check the reliability of conserved domains. Chromosomal locations of LEAs were determined with the information obtained from Gramene database and the physical map was drawn based on their positions. Gene characterization was studied using Gene Structure Display Server (http://gsds.cbi.pku.edu.cn) [44].

### *In silico* characterization of *SbLEA* proteins

The molecular weight (MW), isoelectric point (pI), and GRAVY (grand average of hydropathicity), instability and aliphatic indices were calculated using ProtParam of Expasy tools [45] (http://web.expasy.org/protparam). The NetPhos3.1 software was used to determine the phosphorylation sites within the LEA family [46]. The protein subcellular localization of LEA family members was identified by using WoLF PSORT programs (http://wolfpsort.org/) [47]. The putative *trans*-membrane helices were identified by using TMHMM server (http://www.cbs.dtu.dk/services/TMHMM/) [48]. The conserved motif structures of LEA family genes were retrieved by using Multiple Em for Motif Elicitation (MEME) software (http://meme-suite.org/) with default parameters: number of motifs (1–10), motif width of 5–50, and the number of motif sites (5–10) [49]. The putative miRNAs in targeting the *SbLEA* genes were identified using psRNATarget server [50] with default parameters.

### Promoter analysis of *SbLEA* family, phylogenetic analysis, and estimation of synonymous and non-synonymous substitution rates

The 1000 bp genomic sequence upstream of start codon of *SbLEA* genes were examined using PLACE [51] software to check for the presence of *cis*-elements responsible for development, biotic, and abiotic stresses. The NJ phylogenetic trees for LEA protein family of *S*. *bicolor*, *O*. *sativa*, and *A*. *thaliana* were generated using MEGA 6.2 software [52] with default parameters like Poisson correction, pairwise deletion, and bootstrap value (1,000 replicates). Paralogues and orthologues were identified using phylogeny and InParanoid 8 (the orthology analysis software) [53] with default parameters like 0.01 cut off E value, 50 or higher cut off score values. Synonymous and non-synonymous sites and substitution rates of paralogous and orthologous gene pairs were calculated using PAL2NAL software (http://www.bork.embl.de/pal2nal/) [54].

### *In-silico* expression profiling of *SbLEAs*

Expression analysis for the identified *SbLEA* genes was performed using Affymetrix whole-transcriptome *Sorghum* array data accessible from the SorghumFDB [55]. The Genevestigator platform [56] was used to perform the microarray analysis for *SbLEAs* genes under several environmental stresses (drought, salt, heat, and cold) with different samples embedded in the platform. The expression profiles of *SbLEA* genes identified from *Sorghum* array was used for cluster analysis. A heat map of expression profiling was developed by using hierarchical clustering tool embedded in Genevestigator platform [57].

### Plant material and stress conditions

The seeds of *S*. *bicolor* BTx623 variety were sown in pots containing 4.5 kg of black clay soil under glass house conditions at 28/20 ^o^C day/night temperatures. After 40 days, the plants were subjected to drought and salt stresses by treating with 1 liter each of 150 mM mannitol and NaCl individually for 4 h. The cold stress was applied by keeping the plants at 4°C for 4 h and heat stress by exposing the plants to 40°C for 4 h in a growth chamber. The respective controls were maintained under identical conditions. Roots, stems, and leaves were collected and snap frozen immediately in liquid nitrogen and stored at -80°C until further use.

### RNA extraction and qRT-PCR analysis for transcriptional profiling of *SbLEA* genes

The MACHEREY-NAGEL kit was used to isolate the total RNA from roots, stems, and leaves by following the manufacturer’s instructions. The first strand cDNA was synthesized from total RNA (3 μg concentration) using first strand synthesis kit (Thermo Scientific). Gene specific primers were designed by using NCBI PRIMER Blast (www.ncbi.nlm.nih.gov/tools/primer-blast/) [58] and Primer3 software (http://bioinfo.ut.ee/primer3-0.4.0/) [59] with the default parameters: 57–60°C annealing temperature, 18–22 bp primer length, 50–55% GC contents, and 80–140 bp amplicon length (S1 Table). The SYBR Green Master Mix (2X) (Takara) was used according to the manufacturer's recommendations. Two biological duplicates with three technical replicates were taken for qRT-PCR analysis in Mx3000p (Agilent Technologies) with the following thermal cycles: 1 cycle at 95 ^o^C for 10 min, followed by 40 cycles alternatively at 95 ^o^C for 15 sec and 60 ^o^C for 1 min. The amplicon dissociation curves were recorded with fluorescence lamp after 40^th^ cycle by heating from 58 to 95 ^o^C within 20 min. Transcript levels of *SbAcp* and *SbEP-F* genes were used as internal controls [60]. Relative gene expressions were calculated by employing Rest software [61] and average values are represented. Statistical significance of the expression values was determined by using t-test.

## Results

### Identification, chromosomal localization and gene structure analysis of *SbLEA* genes

**Fig 1:**
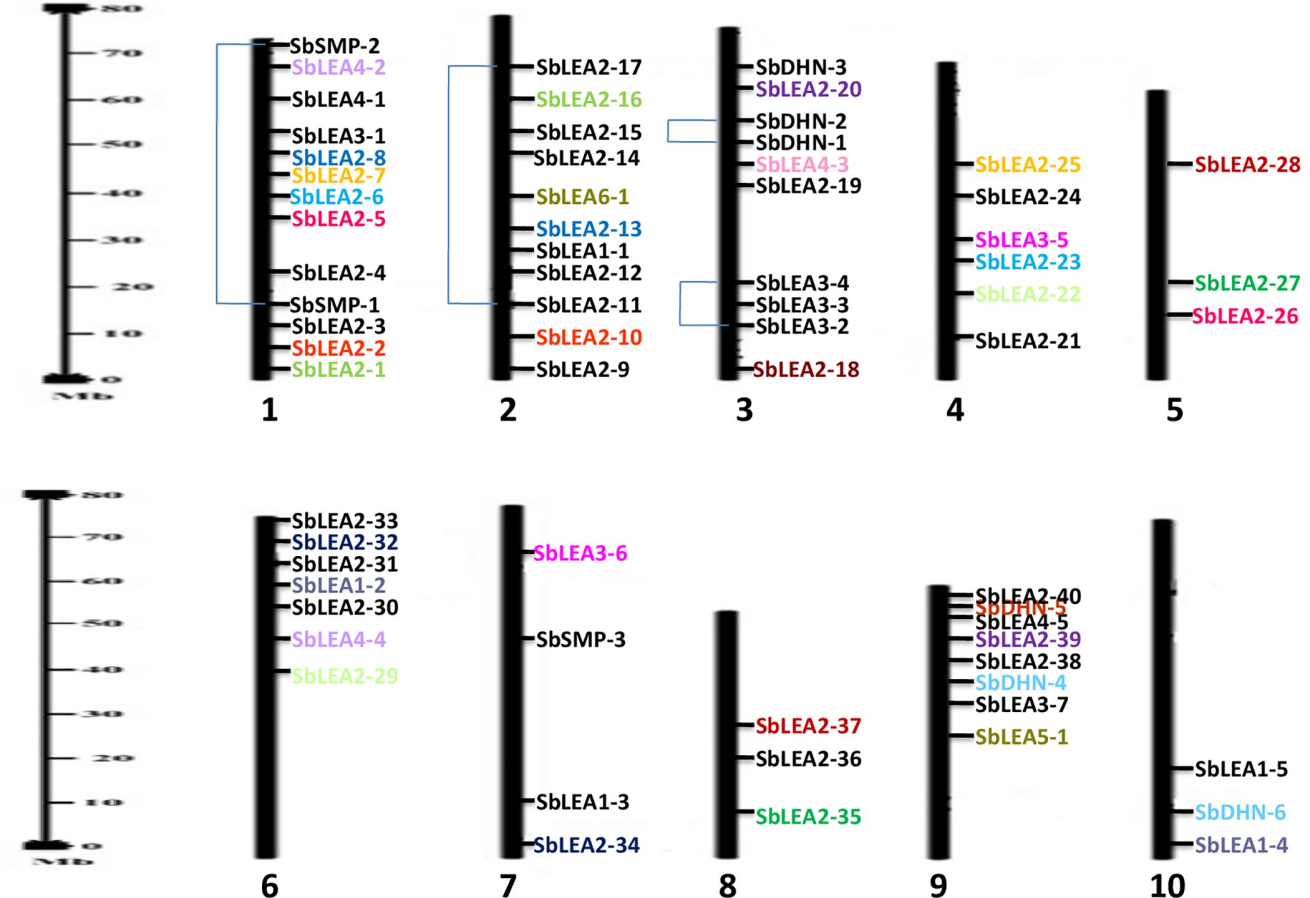
Chromosomal distribution of LEA genes in *Sorghum*. Duplications are illustrated by different colors (Segmental) and regional duplications are linked with line.

**Fig 2:**
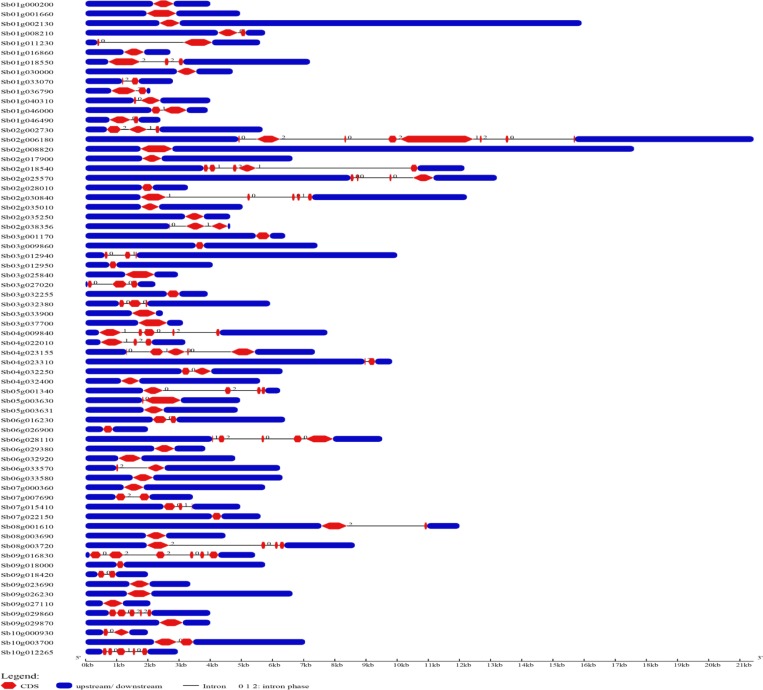
Distribution of exons, introns, upstream and downstream regions in *SbLEAs*.

A total of 68 LEA genes were identified in the genome of *S*. *bicolor* based on rice and *Arabidopsis* LEA homologs. Their reliability was checked for the presence of conserved domain using SMART and MOTIF tools. The genes are grouped into 8 sub-families like LEA 1–6, dehydrins, and SMP based on their conserved domains and Pfam nomenclature. Among all the families, *SbLEA2* was found as the largest family with 40 genes (*SbLEA2-1* to *SbLEA2-40*), followed by *SbLEA3* with 7 genes (*SbLEA3-1* to *SbLEA3-7*), and *SbDHNs* with 6 genes (*SbDHN1- SbDHN6*). Both *SbLEA1* and *SbLEA4* families contain 5 genes each, while *SMP* has only 3 members. The smallest families are *SbLEA5* and *SbLEA6* with one member each (Table 1). *SbLEA* genes were distributed on all the chromosomes. Out of 68 genes, 13 genes are localized on chromosome 1; 11 on 2, 10 on 3, 6 on 4, 3 on 5, 7 on 6, 4 on 7, 3 on 8, 8 on 9, and 3 on 10 (Table 1 and Fig 1). All the members of *SMPs* have only 1 intron and 2 exons. A total of 22 genes out of 40 in the group *SbLEA2* lack introns. *SbLEA2-9* showed a maximum of 8 exons. Out of 68 *SbLEA* genes, only one exon was observed in 31 genes, 2 exons in 19 genes, 3 in 6, 4 in 5, and 5 in 5, 6 in 1, and 8 in 1. A total of 22 genes out of 40 in the group *SbLEA2* lack introns (Table 1 and Fig 2).

**Table 1:** Identified *LEA* genes in *Sorghum bicolor* exhibiting family, number of amino acids, chromosomal location, iso-electricpoint (pI/molecular weight (MW), DNA binding domains (DBD), no. of exons, number of transmembrane helices, localization, GRAVY, instability index, and aliphatic index.

Gene	Family	No. of a.a.	Chro	pI/MW	DBD	No. of exons	TMHMM	Locali-zation	GRAVY	Instability index	Aliphatic index
Sb02g018540	SbLEA1-1	428	2	9.83 / 44845.87	211–283	5	0	Chl	-0.309	37.15*	66.57
Sb07g007690	SbLEA1-2	220	7	9.91 / 23382.56	8–80	2	0	Chl	-0.416	42.05	73.05
Sb06g026900	SbLEA1-3	103	6	9.46 / 10673.89	1–73	1	0	M	-0.963	8.54*	38.45
Sb10g000930	SbLEA1-4	223	10	10.12 / 23960.27	78–150	2	0	M	-0.589	56.17	69.42
Sb10g012265	SbLEA1-5	281	10	9.77 / 31235.36	158–230	5	0	N	-0.684	36.24*	63.91
Sb01g000200	SbLEA2-1	221	1	10.13 / 23553.04	93–200	1	1	Chl	0.197	52.95	94.16
Sb01g001660	SbLEA2-2	318	1	4.84 / 35109.79	80–176	1	0	C	-0.357	19.46*	92.20
Sb01g002130	SbLEA2-3	217	1	9.30 / 23286.06	84–186	1	1	Chl	0.178	26.11*	89.45
Sb01g011230	SbLEA2-4	330	1	10.30 / 36381.87	121–150	2	1	Chl	-0.188	39.74*	86.58
Sb01g016860	SbLEA2-5	219	1	8.67 / 23224.06	97–198	1	1	C	0.231	44.87	101.00
Sb01g018550	SbLEA2-6	438	1	10.16 / 47410.50	309–411	3	1	N	-0.216	46.17	83.29
Sb01g030000	SbLEA2-7	208	1	10.01 / 22777.19	78–183	1	1	Chl	0.083	46.67	98.51
Sb01g040310	SbLEA2-8	238	1	9.10 / 25121.05	114–216	2	1	Chl	0.271	41.61	101.60
Sb02g006180	SbLEA2-9	277	2	8.44 / 132054.42	164–262	8	2	P	0.089	38.50*	102.00
Sb02g008820	SbLEA2-10	341	2	4.88 / 38029.21	57–170	1	0	C	-0.268	33.52*	92.26
Sb02g002730	SbLEA2-11	392	2	10.53 / 41660.30	292–390	3	2	P	-0.213	54.06	76.73
Sb02g017900	SbLEA2-12	208	2	7.49 / 22792.34	80–184	1	1	Chl	0.029	33.27*	84.76
Sb02g025570	SbLEA2-13	304	2	9.88 / 32923.95	199–292	4	1	Chl-N	-0.104	60.95	84.18
Sb02g030840	SbLEA2-14	414	2	8.51 / 45711.33	276–384	5	1	Chl	-0.252	42.94	81.06
Sb02g035010	SbLEA2-15	195	2	7.67 / 20621.92	73–174	1	1	Extr	0.378	35.24*	104.41
Sb02g035250	SbLEA2-16	202	2	9.12 / 22614.24	76–179	1	1	Chl	0.000	46.37	89.21
Sb02g038356	SbLEA2-17	382	2	10.52 / 40386.06	282–380	2	2	Chl	-0.069	48.57	82.77
Sb03g001170	SbLEA2-18	152	3	5.09 / 15933.12	44–141	1	0	Chl	0.036	19.60*	98.75
Sb03g025840	SbLEA2-19	312	3	11.11 / 32197.16	132–232	1	1	Chl	-0.057	53.75	73.91
Sb03g033900	SbLEA2-20	257	3	5.09 / 27235.99	134–235	1	1	C	0.158	37.22*	91.13
Sb04g009840	SbLEA2-21	472	4	11.47 / 50217.36	174–280	5	1	C	0.110	54.48	97.80
Sb04g022010	SbLEA2-22	261	4	9.38 / 28284.61	348–451	3	1	Chl	0.015	54.93	88.81
Sb04g023155	SbLEA2-23	624	4	8.46 / 67773.78	124–228	4	1	Chl-M	0.008	40.78	91.96
Sb04g032250	SbLEA2-24	283	4	5.79 / 31029.23	143–241	2	0	N	-1.282	59.66	57.28
Sb04g032400	SbLEA2-25	196	4	9.27 / 21456.68	68–174	1	1	C	0.097	33.05*	86.53
Sb05g001340	SbLEA2-26	367	5	9.66 / 38483.91	103–206	4	1	Chl	-0.090	52.30	86.49
Sb05g003630	SbLEA2-27	404	5	11.48 / 44027.74	218–367	2	1	Chl	-0.326	69.39	77.08
Sb05g003631	SbLEA2-28	214	5	9.25 / 23456.94	76–179	1	1	Chl	0.106	37.24*	102.06
Sb06g016230	SbLEA2-29	221	6	7.68 / 23727.40	72–175	2	1	Extr	0.306	43.77	99.64
Sb06g029380	SbLEA2-30	216	6	10.22 / 23420.03	89–192	1	1	Chl	0.038	38.81*	85.37
Sb06g032920	SbLEA2-31	247	6	9.99 / 27134.46	120–222	1	1	C	-0.066	44.57	90.77
Sb06g033570	SbLEA2-32	211	6	8.84 / 23254.03	80–184	2	1	Chl	0.194	38.15*	96.59
Sb06g033580	SbLEA2-33	219	6	8.40 / 24148.89	79–184	1	1	Chl	0.162	44.76	97.44
Sb07g000360	SbLEA2-34	214	7	9.04 / 23596.58	82–187	1	1	Chl	0.261	40.02	97.48
Sb08g001610	SbLEA2-35	306	8	9.93 / 32613.27	171–276	2	1	Chl	-0.229	60.33	85.85
Sb08g003690	SbLEA2-36	215	8	9.10 / 23218.41	77–189	1	1	Chl	-0.031	40.99	84.79
Sb08g003720	SbLEA2-37	369	8	11.11 / 32197.16	236–339	4	1	Chl	-0.370	53.14	79.57
Sb09g023690	SbLEA2-38	215	9	10.09 / 22720.53	92–196	1	2	P	0.277	52.73	110.56
Sb09g026230	SbLEA2-39	260	9	5.09 / 26993.88	127–228	1	1	C	0.288	28.46*	96.81
Sb09g029870	SbLEA2-40	252	9	9.95 / 25661.54	122–227	1	1	Chl-C	0.302	30.70*	91.47
Sb01g033070	SbLEA3-1	95	1	9.22 / 10359.84	1–93	2	0	Chl	-0.131	49.37	76.11
Sb03g009860	SbLEA3-2	101	3	10.69 / 9984.19	1–95	1	0	Chl	0.118	35.31*	80.89
Sb03g012940	SbLEA3-3	114	3	9.83 / 12087.83	2–88	3	0	Chl	-0.388	35.31*	73.77
Sb03g012950	SbLEA3-4	79	3	9.70 / 8140.33	1–74	1	0	C	-0.375	25.24*	70.89
Sb04g023310	SbLEA3-5	87	4	10.22 / 9186.44	2–84	2	0	Chl	-0.240	48.40	70.00
Sb07g022150	SbLEA3-6	102	7	9.16 / 11177.89	3–98	1	0	Chl	-0.332	49.57	69.31
Sb09g018000	SbLEA3-7	79	9	4.90 / 8676.85	3–72	1	0	M	-0.511	40.05	66.84
Sb01g036790	SbLEA4-1	352	1	6.96 / 37581.12	47–60	2	0	C-N	-1.047	20.42*	52.44
Sb01g046000	SbLEA4-2	351	1	6.67 / 36503.19	225–328	2	1	Extr	-0.906	28.75*	57.21
Sb03g032380	SbLEA4-3	216	3	8.92 / 22223.27	9–57	3	0	N	-0.875	11.34*	43.84
Sb06g028110	SbLEA4-4	494	6	9.25 / 52617.82	217–256	5	0	M	-0.769	31.46*	61.17
Sb09g027110	SbLEA4-5	214	9	8.48 / 22782.15	32–65	1	0	N	-0.911	40.60	50.37
Sb09g016830	SbLEA5-1	281	9	8.19 / 31089.00	145–277	6	1	N	-0.762	50.56	56.62
Sb02g028010	SbLEA6-1	117	2	6.28 / 12163.34	95–105	1	0	N-M	-0.909	44.27	51.71
Sb01g008210	SbSMP-1	268	1	5.87 / 27668.87	23–267	2	0	C	-0.373	44.51	80.56
Sb01g046490	SbSMP-2	283	1	4.77 / 27953.56	22–81	2	0	Chl	-0.284	32.88*	68.94
Sb07g015410	SbSMP-3	175	7	4.94 / 17311.91	29–93	2	0	C	-0.452	49.09	55.43
Sb03g027020	SbDHN-1	277	3	8.99 / 29881.14	81–277	3	0	N	-0.972	49.39	47.62
Sb03g032255	SbDHN-2	188	3	5.37 / 19842.84	12–178	1	0	Chl	-0.535	52.57	60.16
Sb03g037700	SbDHN-3	309	3	10.02 / 33162.74	141–279	1	1	N	-0.210	59.30	76.63
Sb09g018420	SbDHN-4	152	9	8.81 / 15399.74	2–152	2	0	N	-1.132	23.87*	32.71
Sb09g029860	SbDHN-5	310	9	9.25 / 34622.60	127–180	4	0	M	-0.323	35.51*	76.81
Sb10g003700	SbDHN-6	388	10	8.50 / 37488.09	293–388	2	0	N	-0.836	20.44*	29.05

(a. a.: amino acids, Chro.: Chromosome, pI: iso electric point; MW: Molecular weight, Chl: Chloroplast, C: cytoplasm, N: Nucleus

P: plastid, M: mitochondria, Extr: Extra cellular, GRAVY: Grand average of hydropathicity, *stable)

### *In silico* characterization of SbLEA proteins

The *SbLEA* family genes encode polypeptides ranging from 79 to 624 amino acids in length. While SbLEA3-3 and SbLEA7 contain 79 amino acids (aa), 624 aa are present in SbLEA2-23. Accordingly, the predicted molecular weights range between 8.14 to 67.77 kDa. Among all, the LEA-2 family members show the highest molecular weights (Table 1). Physicochemical analysis reveal that the theoretical pI values range between 4.77 (SbSMP-2) - 11.48 (SbLEA2-27). A total of 50 out of 68 proteins (73.52%) are of basic in nature, while remaining 18 (26.47%) of them are acidic (Table 1). Likewise, SMP group was found to be the most acidic, and similarly in LEA-2, 6 (15%) were identified as acidic. The instability index ranges between 8.54 (SbLEA1-3) and 69.39 (SbLEA2-27) depending upon the group. Nearly 42.64% of SbLEA proteins have a low instability index (> 40), but the LEA-2 group appears unstable (62.5%). The GRAVY of SbLEA proteins vary between -1.282 (SbLEA2-24) to 0.378 (SbLEA2-15). While most of the LEA proteins are hydrophilic, 24 out of 40 (60%) LEA-2 family proteins appear hydrophobic. But, LEA-1, LEA-4, and SbDHNs are completely hydrophilic in nature. The aliphatic index of SbLEA proteins ranges from 29.05 (SbDHN-6) to 104.41 (SbLEA2-15), and SbLEA-2 exhibits the highest from the rest. Contrarily, SbDHNs show the least aliphatic index. It is found that LEA proteins localise mostly to chloroplast (44.11%), followed by cytoplasm (16.17%), nucleus (14.70%), mitochondria (7.35%), plastid (4.41%), and the rest in extra cellular matrix, cytoplasm-nucleus, chloroplast-nucleus, chloroplast-mitochondria, and chloroplast-cytoplasm as revealed by Wolfpsort tool. Majority of SbLEA-2 group members appear to target to chloroplast (~60%). Proteins in the group SbLEA-1 are ~70 aa residues long, with conserved DNA binding domain, whereas in the case of LEA-2 and DHNs family, proteins are 100 residues long. In LEA-3, they are 90 residues long, but in LEA-4 group, they are the smallest with ~30–40 residues. The putative transmembrane helices were identified by using TMHMM server. Only the SbLEA-2 family proteins contain transmembrane helices, some exceptions being SbLEA4-2, SbLEA5-1, and SbDHN-3 (Table 1).

Majority of the SbLEA proteins phosphorylate at serine and threonine sites and very few of them at tyrosine residue. In case of SMP group members, phosphorylation occurs at threonine. Protein kinase C (PKC) and unsp are the most dominant types present in higher amounts in all the SbLEA proteins. Next to PKC, cdc2, PKA, DNAPK, P38MAPK, and PKG are the most common kinases associated with phosphorylation. The highest number of cdc2 was found in LEA-2 family (S2 Table).

### Conserved motif analysis

**Fig 3:**
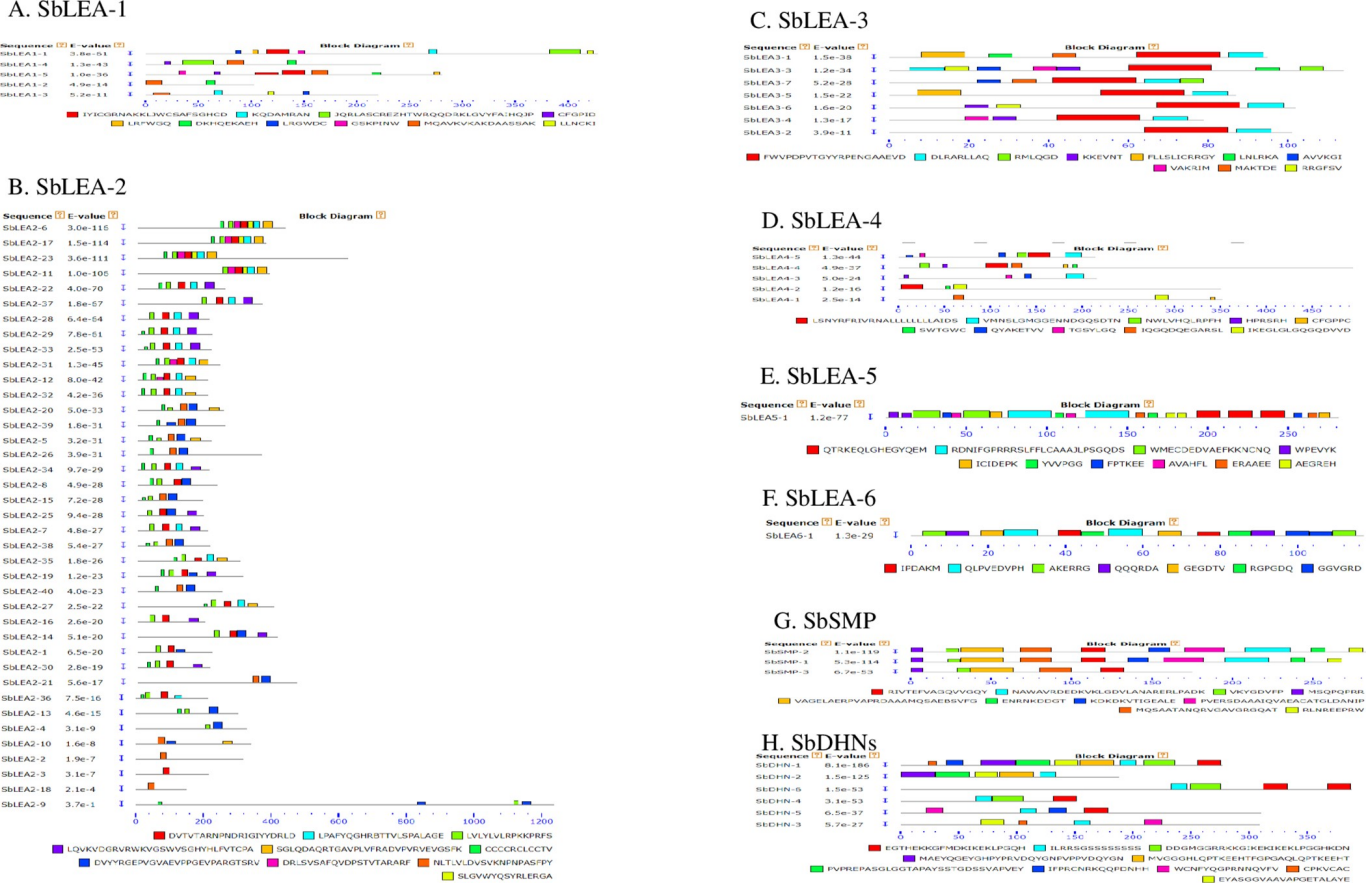
Conserved motif patterns of different SbLEA families. The scale represents the lengths of the proteins and motifs.

Sixty eight SbLEAs did not share high similarity, and each family was submitted to MEME separately and in combination for domain or motif structure analysis. Ten conserved motifs were identified for each family except SbLEA-6, which contains only 7 (Fig 3 and S1 Fig). The paralogs and closely related genes exhibit similar motif compositions. The composition of the motifs is similar in each family but varies among different families. Motif 3 in LEA-1, motif 5 in LEA-2, and motif 5 and 6 in DHNs appeared as the biggest motifs. Fifty four SbLEA proteins exhibit common motifs and motif 1 is the most common and conserved structural motif present in majority of the proteins. Motifs 9 and 10 are the key features of DHN sequences. For recognition of SbDHN proteins, K-segment in motifs 1 and 3, S-segment in motif 2, and Y-segment in motif 4 were used (Fig 3 and S1 Fig). Conserved motifs were not observed in LEA-1, 4, 5, 6, and SMP families. Next to motif 1, motif 3 is the most conserved and located at C terminus. While in LEA-3 group, motif 5 is the most conserved, in LEA-2 family, motif 7 is the structural motif conserved at N terminus (S2 and S3 Figs).

### *In silico* prediction of miRNAs targeting *LEAs*

Our analyses identified that 25 different *SbLEA* genes namely *SbLEA*2-1, 3, 5, 8, 14, 15, 18, 24, 25, 27, 31, 32, 33, 35, 36, 37, 38, and 40, *SbLEA*3-2, 3, 5, 6, and 7, *SbSMP*-3 and *SbDHN*-5 are the targets for 15 different miRNAs. It appears that miRNAs target 18 genes in *SbLEA-2* group, and 5 in *SbLEA-3*. While six miRNAs target *LEA3-5*, 3 of them target *SbLEA2-35* group. Sbi-miR6225, sbi-miR437x, sbi-miR5568, and sbi-miR6220 appear as the most common miRNAs that target *SbLEA* genes and participate in cleavage and translation (S3 Table).

### Promoter analysis of *SbLEA* genes

Promoter analysis revealed that *SbLEA* genes have potential *cis*-regulatory elements, which are further divided into abiotic stress-responsive (DRE, DPBF, MYC, MYB, HSE, LTRE, GT1GM, Cu responsive, Sp1, G-box, and I-box), hormone specific (ABRE, TCA, ERE, etc.), biotic stress-responsive (WBox), development specific (pollen, endosperm specific) and guard cell specific elements (CGCG). The Myb and Myc are the most conserved elements present in all the genes. The salt-responsive elements were observed in *SbLEA-2* family, whereas DRE, and DPBF in all other families and very few of them in *SbLEA-2*. At least one heat shock element (HSE) was identified in all the *SbLEA* families with an exception of dehydrins. ABA-responsive elements (ABRE) and TCA are the most dominant elements present in the highest numbers in all the families. Among all, SMP group exhibits the highest number of ABRE elements (S4 Table).

### Phylogenetic analysis of LEA family proteins

**Fig 4:**
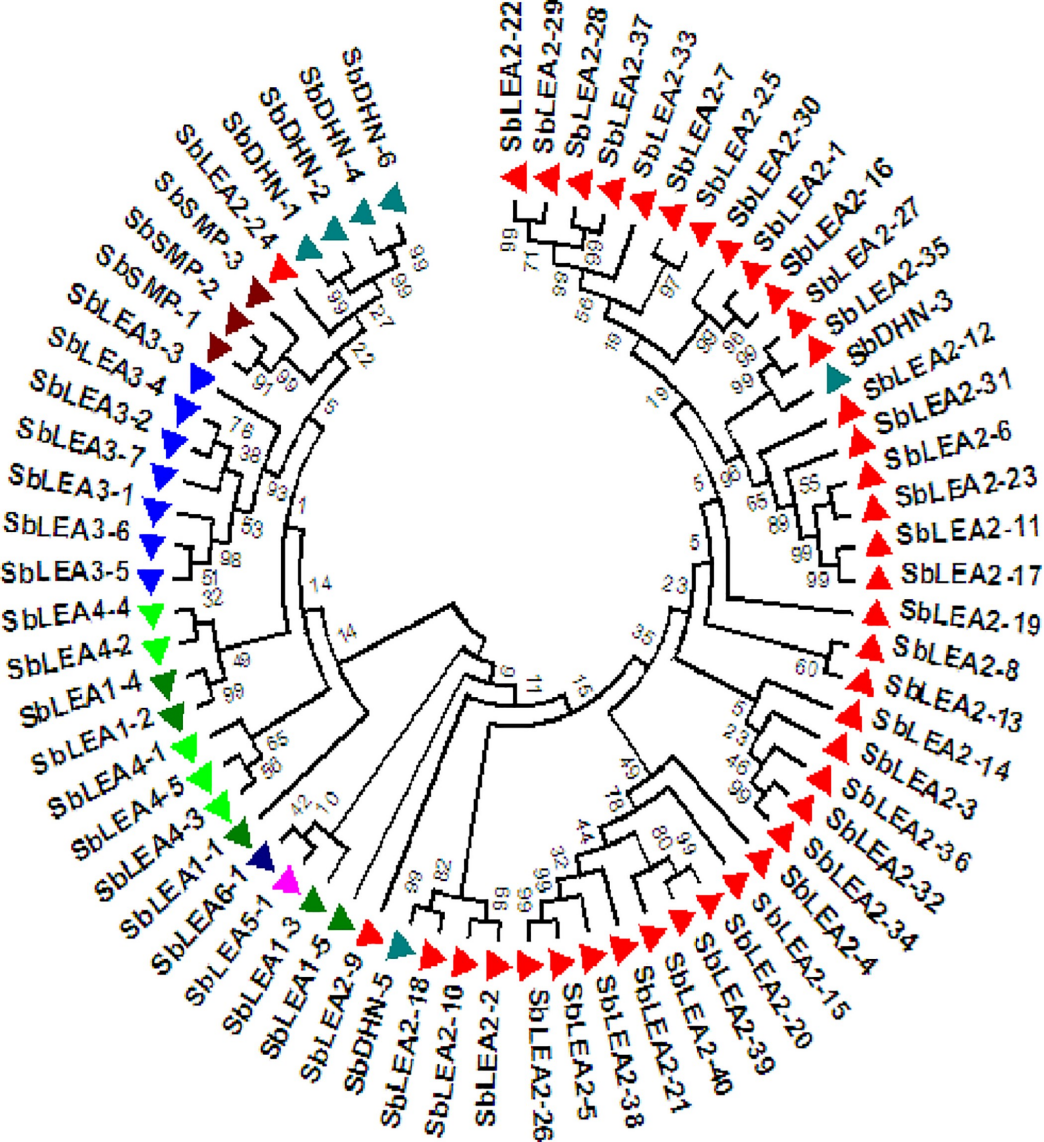
Phylogenetic analysis of 68 *SbLEAs*. *LEA* gene families were classified based on their homology and are distinguished by different colors.

Phylogenetic analysis was carried out for 68 SbLEA proteins to analyse the evolutionary relationships within and between the groups (Fig 4). Different families of *SbLEAs* exhibit high similarity and cluster into 2 major clades (Fig 4). A total of 23 *SbLEA* genes belonging to *LEA-1*, *LEA-3*, *LEA-4*, *SMP*, and *DHNs* form a cluster in clade 1, while the other 45 members of *SbLEA-2* family appear in clade 2. Out of 6 *SbDHNs*, 4 form a cluster into clade 1 (*SbDHN-1*, *2*, *4*, and *6*), and remaining 2 into clade 2 (*SbDHN-3* and *5*). The *SbLEA1-1*, *2*, and *4* are grouped into clade 1, whereas *SbLEA1-3* and *5* into clade 2. Among the 22, 4 regional paralogs were noticed within *SbLEAs*. On the other hand, *SbLEA2-11/13* on chromosome 2, *SbLEA3-2/4* on chromosome 3, *SbSMP-1/2* on chromosome 1, and *SbDHN-1/2* on chromosome 3, and 18 appear as segmental duplications (Figs 1 and 4). *SbLEA-2*, the most dominant group present in *Sorghum*, shows 13 paralogs. *SbLEA-3*, *4*, and *DHNs* show two paralogous events each, while *SbLEA-1* and *SMP* exhibit one event (Fig 4 and Table 2).

**Table 2:** Non-synonymous to synonymous substitution ratios of *LEA* paralogs.

*SbLEA* Paralog gene 1	Chromosome	*SbLEA* Paralog gene 2	Chromosome	No. of Non -synonymous sites (N)	No. of Synonymous sites (S)	Non -synonymous substitution rate (d_N_)	Synonymous substitution rate (d_S_)	d_N_/d_S_
*SbLEA 1–2*	7	*SbLEA 1–4*	10	258.8	50.2	15.4865	0.1826	89.7904
*SbLEA 2–1*	1	*SbLEA 2–16*	2	479.9	126.1	13.3661	11.6073	1.1515
*SbLEA 2–2*	1	*SbLEA 2–10*	2	760.6	193.4	3.4436	10.9315	0.3150
*SbLEA 2–5*	1	*SbLEA 2–26*	5	599.5	57.5	8.5711	59.1966	0.1448
*SbLEA 2–6*	1	*SbLEA 2–23*	4	1017.0	297.0	12.4076	15.0284	0.8256
*SbLEA 2–7*	1	*SbLEA 2–25*	4	448.6	136.4	13.2224	12.2685	1.0778
*SbLEA 2–8*	1	*SbLEA 2–13*	2	561.3	152.7	4.2488	19.0291	0.2233
*SbLEA 2–11*	2	*SbLEA 2–17*	2	904.8	241.2	7.8820	0.0796	99.0000
*SbLEA 2–18*	3	*SbDHN-5*	9	352.7	103.3	3.2717	7.5653	0.4325
*SbLEA 2–20*	3	*SbLEA 2–39*	9	709.7	61.3	7.0826	81.5697	0.0868
*SbLEA2-22*	4	*SbLEA2-29*	6	532.5	130.5	3.2778	6.4948	0.5047
*SbLEA 2–27*	5	*SbLEA 2–35*	8	692.1	225.9	17.1860	0.1736	99.0000
*SbLEA 2–28*	5	*SbLEA 2–37*	8	533.7	108.3	12.2443	16.7231	0.7322
*SbLEA 2–32*	6	*SbLEA 2–34*	7	569.0	64	2.3045	5.0625	0.4552
*SbLEA 3–2*	3	*SbLEA 3–4*	3	176.5	60.5	15.0063	7.1437	2.1006
*SbLEA 3–5*	4	*SbLEA 3–6*	7	209.5	51.5	1.8858	58.2069	0.0324
*SbLEA 4–2*	1	*SbLEA 4–4*	6	884.7	168.3	9.9716	28.9213	0.3448
*SbLEA 4–3*	3	*SbLEA 4–5*	9	505.7	136.3	4.3677	0.0441	99.0000
*SbLEA 5–1*	9	*SbLEA 6–1*	2	285.7	65.3	6.5686	0.0663	99.0000
*SbSMP-1*	1	*SbSMP-2*	1	685.0	119.0	1.0265	81.9485	0.0125
*SbDHN-1*	3	*SbDHN-2*	3	436.0	128.0	13.4524	11.4594	1.1739
*SbDHN-4*	9	*SbDHN-6*	10	362.1	93.9	2.9235	51.8799	0.0564

**(**d_N_ / d_S_ >1 = Positive or Darwinian Selection (Driving Change); d_N_/d_S_ <1 = Purifying or Stabilizing Selection (Acting against change); d_N_/d_S_ = 1 Neutral Selection **)**

**Fig 5:**
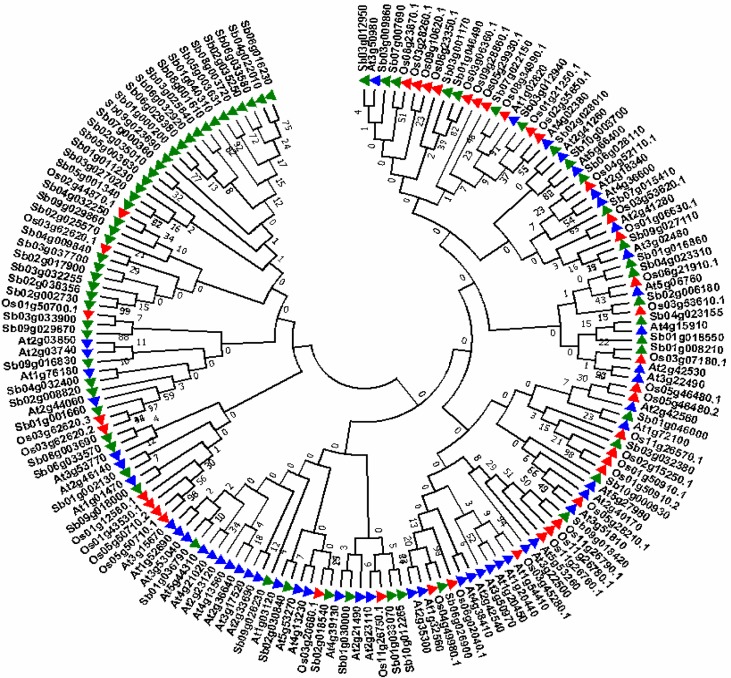
N-J phylogenetic tree showing the relationship between LEA proteins in *Oryza*, *Arabidopsis* and *Sorghum*.

To know the evolutionary relationship and find ortholog pairs, another phylogenetic tree was constructed with *Arabidopsis* and *Oryza* (Fig 5). In this, LEA proteins are grouped into 2 clades, while *LEA-2* family of *Sorghum*, *Oryza* and *Arabidopsis* fall into clade 2, others into clade 1. Thirty eight out of 68 from *Sorghum*, 9 out of 39 from rice, and 7 out of 51 from *Arabidopsis* fall into clade 2, but *SbLEA-2* family appears as the most dominant group. A total of 11 paralogs each are observed in *Sorghum* and *Arabidopsis*, but only 7 in *Oryza*. The *SbLEA* shows 12 orthologs with *Arabidopsis* and 13 with *Oryza*. The *Oryza* and *Arabidopsis* share only six orthologs among them (Fig 5 and S5 Table). From the InParanoid, the orthology analysis of *SbLEAs* exhibits ortholog relationship with *Setaria*, *Oryza*, *Hordeum* and *Brachypodium* (S6 Table).

### Estimation of non-synonymous and synonymous substitution rates of LEA

The non-synonymous (d_N_) versus synonymous (d_S_) substitutions (d_N_/d_S_) were estimated for *SbLEA* genes which show duplication events within *Sorghum* as paralogs (Table 2). The paralogous events, exhibit divergence substitution rates. While the number of synonymous sites (S) ranges between 50.2 (*SbLEA1-2/4*) and 297 (*SbLEA2-6/23*), it ranges between 176.5 (*SbLEA3-2/4*) and 1017 (*SbLEA2-6/23*) in non-synonymous sites (N). In contrast, synonymous substitution rate (d_S_) ranges between 0.0441 (*SbLEA4-3/5*) and 81.9485 (*SbSMP-1/2*), and non-synonymous (d_N_) between 1.0265 (*SbSMP-1/2*) and 17.1860 (*SbLEA2-27/35*) (Table 2). Most of the paralogs *d*_N_/*d*_S_ were found to be below <1 (Table 2). The paralogous synonymous and non-synonymous substitution calculations were extended to orthologous *LEA* gene pairs between *Arabidopsis*, *Oryza* and *S*. *bicolor*. Out of 25 orthologs, *Sorghum* shows 12 events with *Arabidopsis* of which 4 duplications share same chromosomes (Sb01g046000/At1g72100 on chromosome 1; Sb02g028010/At2g41260 on chromosome 2; Sb03g012950/At3g50980 on chromosome 3; and Sb04g023155/At4g15910 on chromosome 4). No such events were observed in *Oryza*. Only 5 orthologs of *Arabidopsis* show d_N_/d_S_ substitution ratios with 99.00. Of the 13 ortholog pairs of *Sorghum* and *Oryza* exhibit d_N_/d_S_ ratios, 5 events show 99, while the remaining vary from 0.0273 to 21.2957 (S5 Table). The orthology analysis of *SbLEAs* with *Oryza*, *Setaria*, *Brachypodium* and *Hordeum* shows that majority of them exhibit Darwinian selection, and the d_N_/d_S_ ratio is greater than 1 (S6 Table).

### Microarray-based gene expression profiling in different tissues and different developmental stages under abiotic stress conditions

**Fig 6:**
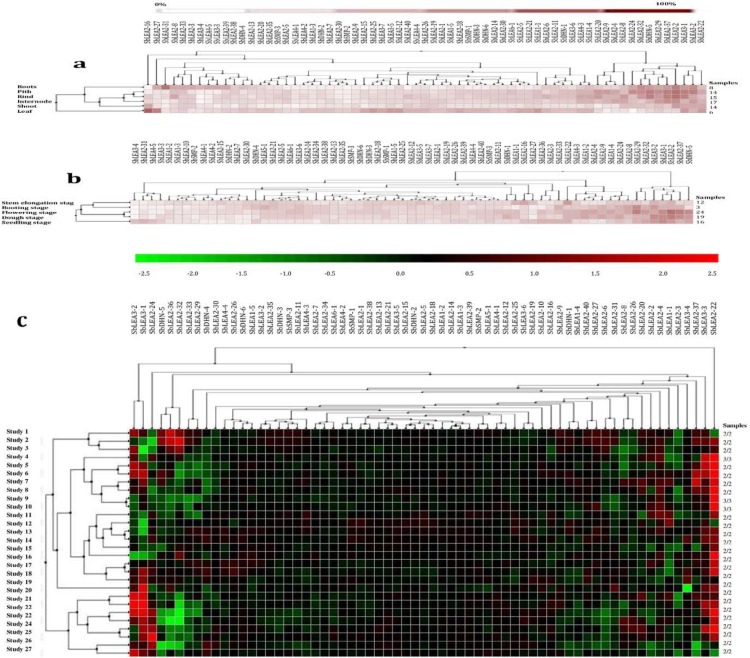
**Digital expression analysis of *SbLEA* genes a) in different tissues; b) in various developmental stages; c) under diverse abiotic stress conditions**. Colour scale represents % expression, down and upregulation.

Of the 68 sorghum *SbLEAs*, microarray data for 65 *SbLEA* genes were available on the Genevestigator platform, these were further utilized for expression analysis. Expression of these 65 *SbLEA* genes in six tissues (roots, pith, rind, internode, shoot, and leaf) was analyzed under normal and abiotic stress conditions using microarray data (Fig 6A). The expression level was higher in root, pith and in the leaf tissues.

The expression profiles of *SbLEAs* genes were analyzed at five different development stages, including stem elongation, booting, flowering, dough, and seedling. *SbLEA* genes were found expressed in all developmental stages (either up-regulated or down-regulated) (Fig 6B). However, the expression of *SbLEA* genes in the booting and flowering stages demonstrated a slightly different pattern, particularly *SbLEA-2* members displayed the dominant expression profile compared to other developmental stages. High expression of *SbLEA* genes during booting and flowering stages might have been caused by booting-related cellular deteriorations, leading to substantial metabolic or physiological changes that significantly affect the overall regulation under abiotic stresses.

Hierarchical clustering based on the above expression profiles of individual *SbLEA* genes under various abiotic stress conditions allowed grouping of the 65 *SbLEA* genes into two major clusters. One of these clusters contained the only *SbLEA2-22* gene which shows very high up-regulation under different stress conditions. The remaining *SbLEA* genes were distributed among other sub-clusters of the second major cluster (Fig 6C). The heat map of different *SbLEA* genes following abiotic stresses showed significantly altered expression (either up-regulation or down-regulation) up to 2.5-folds (Fig 2). Members of the *SbLEA-2* (*SbLEA2-22*, *SbLEA2-24*, *SbLEA2-32*, *SbLEA2-33*, and *SbLEA2-37*) were up-regulated under stress conditions. Similarly, *SbLEA3-1* and *SbLEA3-2* members were up-regulated under salt, cold, and drought stresses.

### Quantitative expression analysis of *SbLEAs*

**Fig 7:**
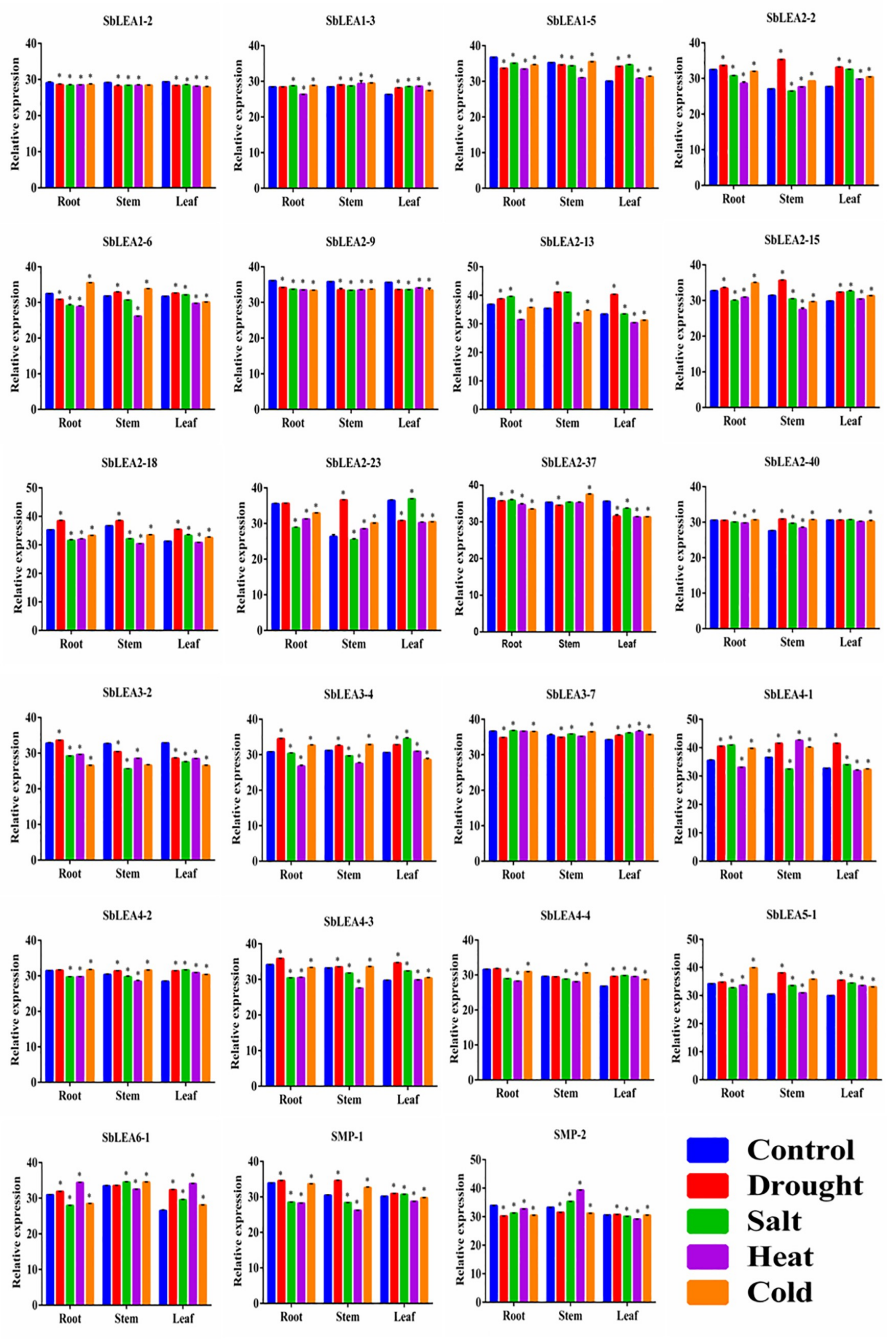
The relative expression values of *SbLEA* genes in roots, stems and leaf tissues under drought, salt, heat and cold stress. Error bars indicate ± SD. * indicate significant differences calculated by t-test (*P ≤ 0.05).

**Fig 8:**
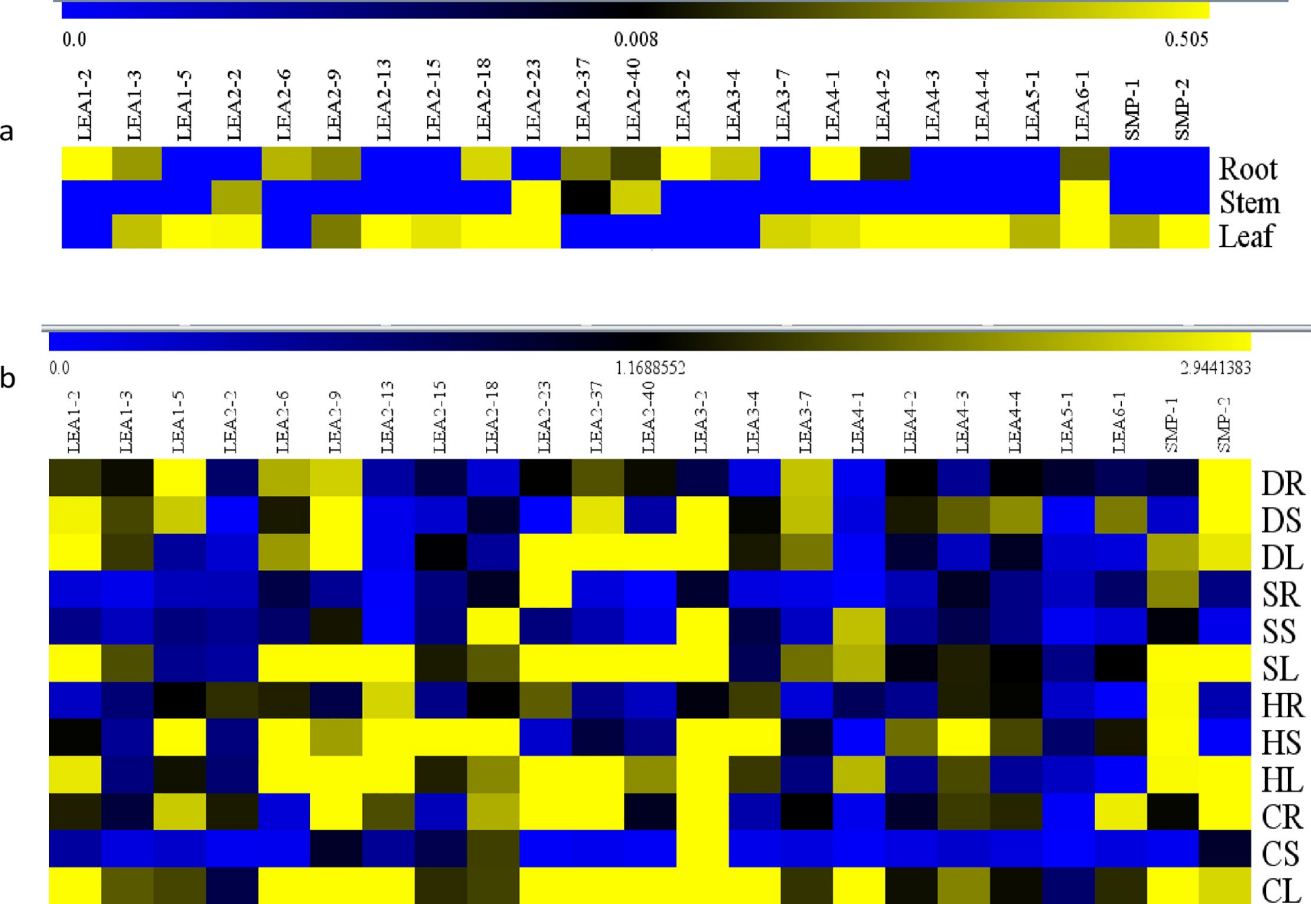
**qRT-PCR expression patterns of *SbLEAs* a) tissue specific expression of *SbLEAs* in roots, stems, and leaf tissues; b) transcriptional expression analysis of *SbLEA* genes in roots, stems, and leaf tissues under drought, salt, heat and cold stresses.** (DR: Drought Root, DS: Drought Stem, DL: Drought Leaf, SR: Salt Root, SS: Salt Stem, SL: Salt Leaf, HR: Heat Root, HS: Heat Stem, HL: Heat Leaf, CR: Cold Root, CS: Cold Stem, CL: Cold Leaf). The expression represents log2 values.

To investigate the differential gene expressions in vegetative tissues of *Sorghum*, a systematic analysis of quantitative real-time (qRT)—PCR was carried out for a group of 23 *SbLEA* genes. qRT-PCR expression analysis of 23 *SbLEA* genes in different tissues under drought, salt, heat, and cold stresses reveals their comprehensive roles in stress tolerance mechanism, as well as in growth and development. The differential expression patterns in roots, stems, and leaves are shown in the Figs 7 and 8A. Most of the *LEA* genes exhibit the highest expression levels in stem tissues (*SbLEA1*-*5*, *2–9*, *2–13*, *2–18*, *2–37*, *3–7*, and *4–1*) (Fig 8A). Compared to leaf and stems, root tissues show lower expression values under the stress conditions. The *SbLEA3-2* show the highest expression levels in leaf tissues under salt (82-folds), cold (434-folds), and drought stresses (52-folds), and in stem under salt stress (445-folds). On the other hand, *SbLEA2-23* show several-folds increase in leaf tissues under drought (191.78-folds), and cold (340.93-folds), whereas in roots under heat stress (369.64-folds). Surprisingly, members of *SbLEA-2* (the major family) display high expression under all stresses in leaves compared to other tissues, the *SbLEA2-37* exhibit 48.95-folds in drought-exposed and 99.27-folds in cold-treated leaves. Expression of *LEA1-5* in roots is better under drought stress (11.28-folds), and in stems under cold stress (15.06-folds). The *LEA-4* family members exhibit the highest expression in stems under stress compared to other tissues; the *LEA4-3* exhibits the highest expression in stems under cold stress (39.4-folds). Interestingly under heat stress, expression of majority of the *SbLEA*s was high in roots, the *SbLEA1-2* exhibits 14.22-folds, *SbLEA2-9* 28.24-folds, *SbLEA2-23* 369.64-folds, *SbLEA2-37* 111.43-folds, and *SbLEA3-2* 133.43-folds. Expression of *SMP-2* is high in root tissues under drought (16.99-folds) and cold (10.85-folds) stresses, but the leaf tissues display high activity (11.65-folds) under salt stress (Figs 7 and 8B and S7 Table).

## Discussion

Genome-wide analysis of *Sorghum bicolor* for *LEA* genes reveals 68 *SbLEAs* that belong to 8 families. Similar studies in other plant species showed different number of *LEAs*; 23 in *Phyllostachys* [62], 27 in tomato [26], 29 in potato [25], 30 in *Prunus* [24], 32 in maize [63], 34 in rice [23], 36 in soybean [64], 51 in *Arabidopsis* [7], 53 in poplar [21], 61 in *Cucumis melo* (melon) and 73 in *Citrullus lanatus* (water melon) [65], 72 in sweet orange [66], 79 in cucumber [67], 108 in *Brassica* [20], 136 in *Gossypium arboreum*, 142 in *G*. *raimondii*, and 242 in *G*. *hirsutum* [68]. It is puzzling to note that the number of *LEA* genes is very large, abundant and diversely distributed across different taxa. The abundance perhaps indicates their conservative role under abiotic stress conditions as well as during growth and development. It is interesting to observe that aquatic plants have less number of *LEAs* because they do not suffer from drought stress. Thus, the present and previous research findings are consistent with the results of Kamisugi and Cuming [69] regarding the wider distribution and function of LEA proteins in terrestrial plants. Generally, the *LEA* families with close taxonomic relationships exhibit the same number and distribution of genes. However, the number of the *LEA* genes varies in *Sorghum*, maize, and rice. This occurrence may be due to the evolutionary variations of the whole genomes and wide changes in the environment. Comparison of *SbLEAs* with rice and maize show divergence signals which are associated with selected traits and are functionally stress-responsive. This indicates that stress adaptation in maize is possible by evolution of protein coding sequences [70]. The divergence of *LEA* families in *Zea* and *Oryza* occurred due to evolutionary changes, the large number of *LEA* genes and evolution of *LEA-2* family members may be meant for adaptation of *Sorghum* to stress conditions. The LEA family proteins are further classified into 8 subfamilies among all the crops based on their conserved domain and phylogenetic tree analysis. But, *Arabidopsis* holds an extra subgroup named as AtM [7]. In *Sorghum*, the most dominating *LEA-2* family has the highest number of genes (58.8%), but dicots such as *Arabidopsis* (codes for 35%), *Populus* (49%) *and Brassica* (23%) are rich in LEA-4 members [7, 20, 21]. Similarly, *DHNs* and *SMP* groups also show variations among monocots and dicots. *Arabidopsis* consists of 10 *DHNs*, and 6 *SMPs* [7], *Oryza* 8 *DHNs*, 5 *SMPs* [23], *Brassica* 23 *DHNs*, 16 *SMPs* [20], and *Sorghum* 6 *DHNs* [71], and 3 *SMP* genes. The expansion of gene family depends on segmental, tandem duplications, and transposition events [72]. In the present study, 22 paralogs were observed including 4 regional duplications, and 13 paralogous pairs (*SbLEA-2* family) with segmental duplication events. This indicates that segmental and tandem duplications are responsible for *SbLEA* gene family expansion [20, 26]. Lan et al. [21] pointed out that stress-responsive genes generally contain very less number of introns. In the present study, 45.58% of *LEA* genes lack introns (especially 55% of genes in the major *SbLEA-2* group) and 27.94% hold one intron. Similar results were recorded in *Brassica* [20]. This supports the earlier view that introns delay the gene expression and extend the transcript length, which results in an additional burden on the process of transcription [73].

Filiz et al. [22] and Altunoglu et al. [62] pointed out that LEA4, LEA5, and LEA6 group proteins are acidic while most of the LEA proteins are basic in nature. Present study shows that 73.52% are basic in nature, but 85% of proteins from SbLEA-2 are basic thus corroborating the earlier findings. In contrast, SMPs are found to be acidic in nature which is in agreement with the findings of Liang et al. [20] in *Brassica*. The grand average of hydropathy values of SbLEA proteins are highly hydrophilic, except SbLEA-2 family. Previous studies report only one or two proteins with hydrophobicity [7, 20], while 85% of SbLEA-2 group proteins are hydrophobic, similar to cotton LEA2 members [68]. Hydrophilic nature and high net charge are the characteristic features of LEAs [74], which makes them disordered, and act like molecular chaperones under stress in plants [75].

Instability index shows majority of the SbLEA proteins are stable like that of SiLEAs as noticed by Cao and Li [26]. LEA proteins are not transmembrane proteins [76] and are located in mitochondria, chloroplasts, nucleus, and cytoplasm. Contrarily, SbLEA-2 family members exhibit transmembrane helices, which are hydrophobic in nature. Detection of transmembrane helices in proteins indicate their expression in subcellular compartments. SbLEA-2 shows high aliphatic index inferring the relative volume occupied by aliphatic side chains like alanine, valine, isoleucine and leucine, which enhance the thermostability of proteins [77]. Majority of the SbLEA-2 family members are localized in chloroplasts, like in cotton [68]. The wide distribution within subcellular compartments leads to interaction with cellular membranes under stress and establish protective mechanism for stress tolerance [15].

Generally, the diversity of structure and conserved motifs cause the evolution of multigene families [78]. It is the amino acid composition that causes disordered structure in LEAs [79]. Our analysis revealed that SbLEA proteins show group-specific conserved motifs. Identical results were reported earlier for LEA proteins in *Arabidopsis* [7], *Prunus* [24], poplar [21], *Solanum* [26], maize [63], *Brassica* [20], and cotton [68]. Specific conserved motifs and their number indicate that they are evolved from the gene expansion within their specific families, and the motif compositions vary from one family to the other. While glycine-rich regions are noticed in AtLEA-2, other LEA members are rich in lysine [7]. But, conserved motifs in SbLEA-2 family are rich with cystine and lysine in contrast to hydrophilins that lack tryptophan and cysteine [80]. The intrinsically disordered proteins which are small in size play several important roles in cells that help in structural flexibility, binding of DNA, RNA, proteins, macro molecules, and membrane proteins to protect and maintain the cellular stability under stress [75, 81, 34]. Phosphorylation helps LEA and dehydrin proteins in binding to calcium, iron and other divalent cations [82, 83]. Phosphorylation of YnSKn type DHNs by PKCs, and SKn DHNs by CK2s, maintains the activity of DHNs conferring tolerance to stress. Eriksson and Harryson [84] and Nagaraju et al. [71] pointed out that such phosphorylation enhances the membrane binding activity of DHNs.

Micro RNAs (miRNAs) are the large group of small, noncoding regulatory elements, which play pivotal roles in gene regulation by disturbing the transcripts of genes and mediate the plants adaptation under abiotic stress [85–87]. For example, expression of rice miR319a in creeping bent grass confers tolerance against salt and drought stresses [88]. Also, salt stress alters the expression of miR396c and miR394 [89]. Sb-miR437, found in majority of *SbLEA* genes has also been identified earlier in *Oryza*, maize, and sugarcane but absent in *Arabidopsis* and *Populus*. This suggests that miR437 is monocot specific [90]. *Sorghum* miRNAs may target transcription factors like SPB, zinc finger, WRKY, WD-40, NAC, MYB, HSFs, GRAS, ARFs, and bHLH families [91], which play important roles in growth, development, metabolism, biotic and abiotic stresses [92–94].

Present study identifies several abiotic stress-responsive elements, hormone specific, development specific, and biotic stress-responsive elements, as also noticed in other crop plants [26, 68]. The *cis*-elements responsive to phytohormones increase the plants potentiality to survive under environmental changes. It is known that ABRE play an important role in ABA signalling and abiotic stress tolerance. Similarly, DRE/CRT/LTRE (drought responsive/C-repeat/low temperature-responsive) elements enhance the drought, cold and salt-responsive gene expression, by controlling transcription factors like CBF/DREB1 [95, 96]. Multiple CGCG *cis*-elements present in all the *SbLEAs* bind to calmodulin/Ca^2+^ and are responsible for eliciting multiple signaling pathways [97]. *SbLEAs* also contain biotic stress-responsive *cis*-elements; WBOXNTERF3, WBOXATNPR1, and CGTCA that respond to wounds, pathogens and salicylic acid [98, 99]. GT1GMSCAM4 *cis*-elements, rich in GAAAAA, were detected, and play a crucial role in salt and pathogen-induced gene expression and tolerance [100]. The MYB *cis*-acting promoter elements identified in the present study play a key role in the abscisic acid-dependent signaling pathway in response to drought, salt, and cold as pointed out by Li et al. [101]. Identification of wide range of *cis*-elements in the *Sorghum* paralogous gene promoter regions perhaps indicate the variation in expression between paralogous duplicated genes, neo-functionalization or sub-functionalization, which is an important evolutionary mechanism [102]. The presence of these *cis*-elements in *SbLEA* genes represent that they play important roles in different stresses.

Based on the phylogenetic analysis, *SbLEA* genes were classified into 8 groups, similar to other plants [7, 20, 68]. While *SbLEA2* is the largest group, *SbLEA5* and *6* represent fewer genes, consistent with *Arabidopsis* [7]. Interestingly, *LEA6* group is absent in rice [23]. The present study revealed 25 ortholog gene relationships with *Arabidopsis* and *Oryza*. Generally, *Sorghum* exhibits relationship with *Oryza*, being the common monocot ancestor, but the present study reveals that *S*. *bicolor* LEA proteins are phylogenetically close to *Arabidopsis* also. The phylogenetic tree depicts common evolutionary origin of *LEA-1*, *3*, *4*, *5*, *6*, and *SMP* [6], which is consistent with potato and cotton [25, 68]. Genome-wide analysis in few plants reveals the differences among LEAs in monocots and dicots. In dicots, *LEA4* and *DHNs* are the most abundant [7, 20, 26], but analysis of *Sorghum* reveals LEA2 is a big, atypical, hydrophobic group. A recent study in rice and poplar reports higher number [66]. The phylogenetic analysis reveals that whole genome duplication contributes to expansion of *SbLEA* family. Indeed, rice (monocot ancestor) genome contains 34 *LEA* genes [23], and the whole genome duplication event is expected to generate 68 genes as seen in *Sorghum*. Similar results were observed in *Arabidopsis*, *Brassica*, and cotton also. Out of a total of 22 paralogous duplication events, 1 segmental and 4 tandem duplications are observed in *Sorghum*. As pointed out by Salih et al. [103], the abundance of LEA proteins mainly occur through segmental duplication events during evolution, similar to *Arabidopsis*, *Brassica*, and cotton. It is known that the synonymous (d_S_) and nonsynonymous (d_N_) values reveal the selective pressure on *SbLEA* duplicated genes. While greater than 1 d_N_/d_S_ value indicates positive selection, less than 1 functional constraint, and equal to 1 neutral selection [104]. The d_N_/d_S_ ratio analysis of *SbLEA* 22 paralogous pairs reveal that only 11 events had ratios of which one shows more than 1, and remaining very low values, similar to *Brassica* [20], melon [65], and cotton [68]. This infers that during evolution, the purifying selection influences the *SbLEA* genes and specifically *LEA2* shows conserved structures and functions under selective pressure [105].

Gene expression analysis provides new insights into their function [106, 107]. Microarray data from the databases show high expression of *SbLEA* genes in different tissues. This indicates that abiotic stresses and/or high metabolic activity generally lead to up-regulation of *SbLEA* genes in different tissues in a tissue-specific manner. These results agree with the results of quantitative real-time expression analysis carried out for a set of *SbLEA* genes in the present study. *SbLEA* gene expressions in different tissues exhibit variations, which reveal their role during growth and development. Both *SlLEA9* and *SlLEA23* show high expression levels in tomato flower buds, suggesting their roles in reproductive development [26]. The At5g27980 regulates pollen germination and tube growth due to its abundant expression in the mature pollen [108, 109]. Expression of *ZmLEA3* group in root, stem, and leaf tissues also suggests their role in growth and development [63]. Present study shows abundant expression of *LEA2* group genes in vegetative tissues, akin to cotton *LEA*s [68]. Majority of the *SbLEAs* are expressed in leaf tissues, consistent with the observations of Liang et al. [20] in *Brassica*. Native expression of paralogous genes in different tissues implies distinct divergence and evolution of duplicated genes for different functions during plant growth and development. *SbLEA* genes expression was further assessed under drought, salt, heat, and cold in different tissues, which gives new insights into their critical roles under abiotic stress conditions. These results show significant changes in expression levels under diverse stresses implying their association with stress tolerance. They act as molecular chaperones, protect, stabilize, prevent aggregation and denaturation of proteins under stress conditions [110]. Among different tissues, roots are first affected under many abiotic stresses [111], followed by leaves. Leaves wilt or become chlorotic and lead to disruption of photosynthesis and yield losses [112]. The paralogs also show expression variations similar to previous studies by Du et al. [24]. Expression of *ZmLEA3* at the transcriptional level was reported under biotic and abiotic stresses and its over-expression in tobacco exhibit tolerance against osmotic and oxidative stresses by participating in protein protection mechanism and by binding to metal ions [36]. Similarly, *SbLEA3-2* upregulates in leaf tissues under all stresses, acting as regulatory gene that participates in stress tolerance mechanism. *SbLEA1-5*, *SMP-1*, *SMP-2*, *LEA3-2*, *LEA4-3*, and many members of the *SbLEA-2* group upregulate in stem under heat, drought, and salt stresses. Over expression of *SiLEA14* enhances abiotic stress tolerance in foxtail millet [113]. While overexpression of tomato *LEA25* enhances salt and chilling stress tolerance in yeast [29], *NtLEA7-3* displays tolerance against cold, drought, and salt stresses in *Arabidopsis* [28]. The *Brassica BnLEA4-1* expressed in *E*. *coli* exhibits tolerance to temperature and salt stresses [32]. The *SbLEA-2* family members, a typical hydrophobic proteins, upregulate under different stresses, and the results are consistent with that of cotton which show high expression under drought stress [68]. The *Medicago MtPM25*, a hydrophobic protein participates in disaggregation of proteins under stress, but unable to protect membranes [114]. Thus, the abundant presence of *LEA-2* genes under stress conditions indicates that they act as key factors in plant adaptation mechanism under diverse environmental stresses.

## Conclusion

A systematic genome-wide analysis resulted in the identification of a total of 68 *LEA* genes in *Sorghum*, which are classified into 8 groups and distributed on all the chromosomes. For the first time in monocots, a typical hydrophobic group *SbLEA2* is identified with large number of genes like that of dicots. Present study helps in understanding the evolution and functions of an important major family *SbLEA2* by functional analysis. It appears that segmental and whole genome duplication plays an important role in their expansion. The gene organization and motif compositions of the *LEAs* are highly conserved which indicate their conserved functional roles. Alongside the abiotic stress-responsive elements, hormone specific, developmental, biotic and other *cis*-elements were identified, indicating their complex regulatory mechanism. Further, the diversified and tissue specific expression profiles provide a further insight into the possible functional divergence in *SbLEA* gene family. The transcriptional profiling under abiotic stress indicates they might play an essential role in stress tolerance. Taken together, present study lays the foundation for further investigations of the specific functions of these *Sorghum LEA* genes, especially *LEA2* family, in other monocots with reference to abiotic stress tolerance.

## Supporting information

S1 Fig MEME identified motif sequences of LEA proteins in *Sorghum*. (TIFF)

S2 Fig Motif distribution of LEA proteins in *Sorghum*. (TIFF)

S3 Fig Web logos of SbLEA proteins conserved motifs. (TIFF)

S1 Table *SbLEA* gene primers used in the gene expression analysis. (DOCX)

S2 Table Types of protein kinases in the phosphorylation of SbLEAs. (DOCX)

S3 Table miRNAs targets *SbLEA* genes. (DOCX)

S4 Table Conserved *cis*-acting elements in *LEA* promoters of *Sorghum*. (DOCX)

S5 Table Non-synonymous to synonymous substitution ratios of LEA orthologs. (DOCX)

S6 Table d_N_/d_S_ ratios of *SbLEA* orthologs between *Sorghum*, *Setaria*, *Oryza*, *Brachypodium* and *Hordeum*. (DOCX)

S7 Table Native and relative expression analysis of *SbLEAs*. (DOCX)
