# Supplementary figures and images for: Genome-scale identification, classification, and tissue specific expression analysis of late embryogenesis abundant (LEA) genes under abiotic stress conditions in Sorghum bicolor L
Source: PLoS One. 2019 Jan 16;14(1):e0209980. doi: 10.1371/journal.pone.0209980 (PMC6335061; doi:10.1371/journal.pone.0209980)

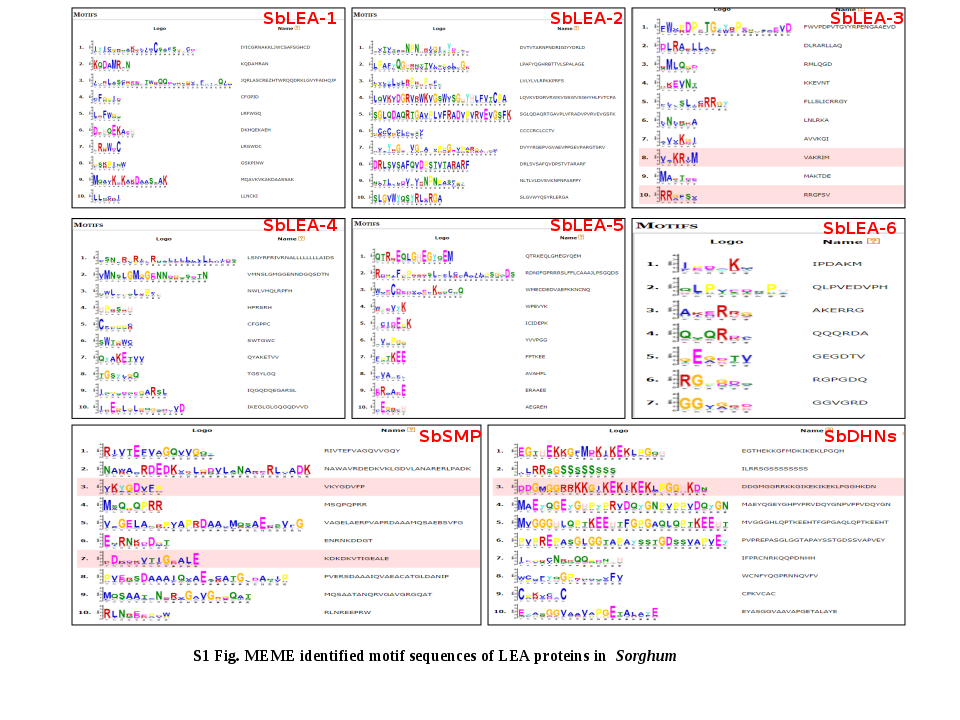

Supplement: S1 Fig — (TIFF) [file pone.0209980.s001.tiff]

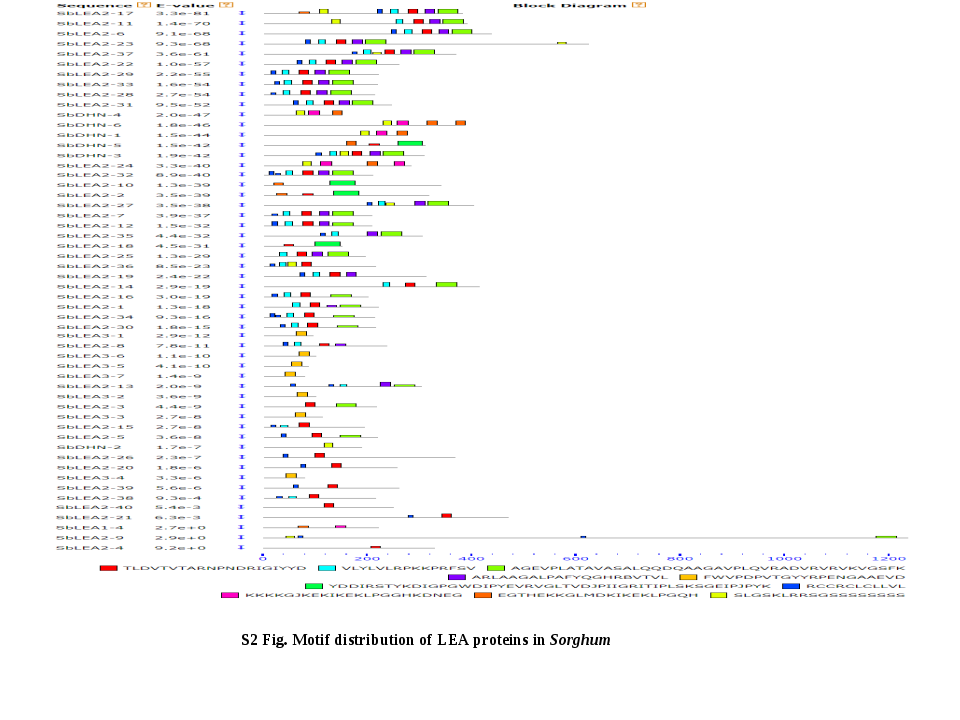

Supplement: S2 Fig — (TIFF) [file pone.0209980.s002.tiff]

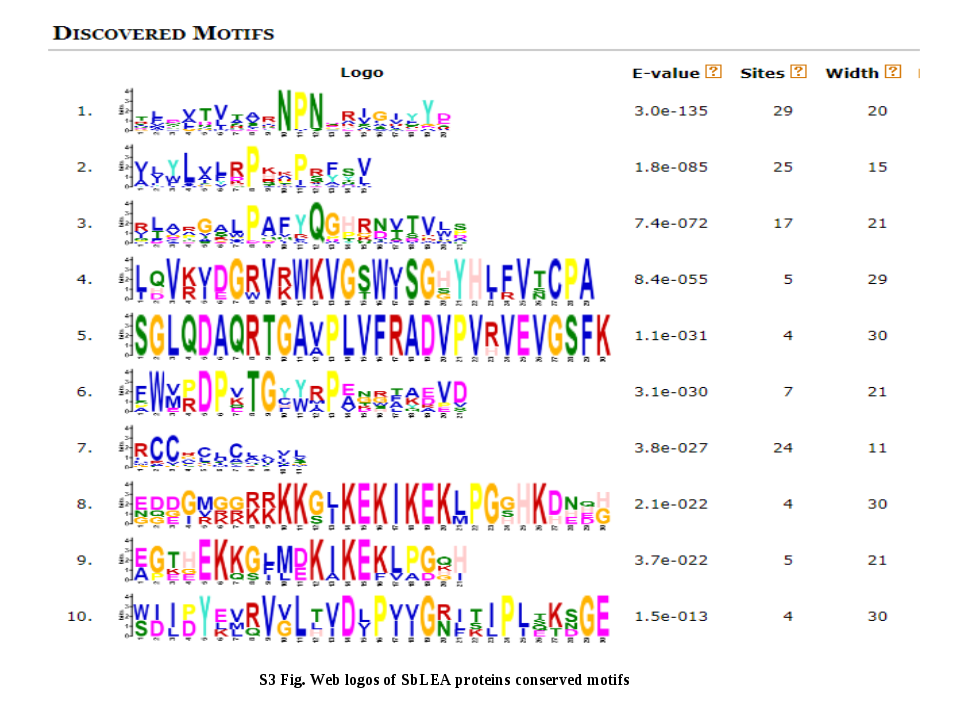

Supplement: S3 Fig — (TIFF) [file pone.0209980.s003.tiff]
