# Supplementary material for: Genome-scale identification, classification, and tissue specific expression analysis of late embryogenesis abundant (LEA) genes under abiotic stress conditions in Sorghum bicolor L
Source: PLoS One. 2019 Jan 16;14(1):e0209980. doi: 10.1371/journal.pone.0209980 (PMC6335061; doi:10.1371/journal.pone.0209980)
Supplement: S1 Table — (DOCX) [file pone.0209980.s004.docx]

| S. No.  **S1 Table.** *SbLEA* gene primers used in the **gene expression analysis** | Gene Name | Primer sequence | Tm | GC | Amplicon  Size |
| --- | --- | --- | --- | --- | --- |
| 1 | SbLEA1-2 | F:CCAATCACGTGGATGGGACT | 60 | 55 | 107 |
|  |  | R: CTTGAACAGCCACGATGACG | 60 | 55 |  |
| 2 | SbLEA1-3 | F:AAGATGCAGGGCAAGACGG | 60 | 57 | 116 |
|  |  | R: TTCTCCTGGTGCTTGTCGG | 60 | 57 |  |
| 3 | SbLEA1-5 | F:CAACGTGTGCCAAAGCTACC | 60 | 55 | 114 |
|  |  | R: CGGAGGACGCTGACATGTTT | 60 | 55 |  |
| 4 | SbLEA2-2 | F:GCGGGTTCATTGACAAGGTG | 60 | 55 | 118 |
|  |  | R: CTGATGTGCGGGATGTGGAT | 60 | 55 |  |
| 5 | SbLEA2-6 | F:CGTGTGACGAGGAAAACGTG | 59 | 55 | 84 |
|  |  | R: CATACTATGACCAGCCGGGA | 58 | 55 |  |
| 6 | SbLEA2-9 | F:CTCAAGGTTGCGATGCTGTG | 60 | 55 | 95 |
|  |  | R: ACCATGACATCGCTGAACCC | 60 | 55 |  |
| 7 | SbLEA2-13 | F:CTACATGGAGATCAGGGTCGC | 60 | 57 | 101 |
|  |  | R: GAAGAGTGTGCGTGGAGTCA | 60 | 55 |  |
| 8 | SbLEA2-15 | F:TTGGGTGGAGTTTCCGACAG | 60 | 55 | 94 |
|  |  | R: CGCCCTGATCTTGATTGTGC | 60 | 55 |  |
| 9 | SbLEA2-18 | F:GACATCGACTACGAGATGCG | 60 | 55 | 120 |
|  |  | R: TCAGAAGATGCTGGAGAGCG | 60 | 55 |  |
| 10 | SbLEA2-23 | F:GCAGATCGGCACAGTTTTGG | 58 | 55 | 110 |
|  |  | R: CTTCGTGGTTTGCCTTGCTC | 59 | 50 |  |
| 11 | SbLEA2-37 | F:TGACCCGTCCACGAAAAAGC | 61 | 55 | 108 |
|  |  | R: CTTTTTCAACCACGCAGGGG | 60 | 55 |  |
| 12 | SbLEA2-40 | F:GGCGACATCGTGATCCACA | 60 | 58 | 84 |
|  |  | R: CGCTGCTCCTTCCACGAAGA | 62 | 60 |  |
| 13 | SbLEA3-2 | F:GAAGATTGGCAGCAGCTTCG | 60 | 55 | 80 |
|  |  | R: GTTGAACATTCACCGCCGTC | 60 | 55 |  |
| 14 | SbLEA3-4 | F:CAAGAAGGAAGTGAACACCGC | 59 | 52 | 96 |
|  |  | R: CTACCTGCTGGGCGATAGTA | 58 | 55 |  |
| 15 | SbLEA3-7 | F:GAGAAGACGGCGTTCTGGGA | 60 | 60 | 111 |
|  |  | R: GAGAAGACGGCGTTCTGGGA | 60 | 60 |  |
| 16 | SbLEA4-1 | F:CAGCACTCACGACACCGATA | 60 | 55 | 90 |
|  |  | AGCGTCAGCTTCTCCTTGAT | 60 | 50 |  |
| 17 | SbLEA4-2 | F:CCAAGACGAACGTCCACGA | 60 | 57 | 83 |
|  |  | R: GGAGATCTTCTCCTTGGCCC | 60 | 60 |  |
| 18 | SbLEA4-3 | F:AAGGACGCGGTGATGAACTC | 60 | 55 | 111 |
|  |  | R: CTAATAGTCCCTGCCAGGCT | 58 | 55 |  |
| 19 | SbLEA4-4 | F:TCTAATCGCCATTGGAGCGG | 60 | 55 | 109 |
|  |  | R: GCCCGCACTATCTAGGGTTA | 59 | 55 |  |
| 20 | SbLEA5-1 | F:TCTGCTGCTGCTGTTGTAGG | 60 | 55 | 80 |
|  |  | R: GTTGCTGGAGCAGTACGAGT | 60 | 55 |  |
| 21 | SbLEA6-1 | F:CATCCCCGTGGCGAAGAC | 60 | 66 | 80 |
|  |  | R: CAACTCCTCCACCACCTCC | 60 | 63 |  |
| 22 | SbSMP-1 | F:ACGAGGACAAGGTCAAGCTC | 60 | 55 | 130 |
|  |  | R: GTCATGTCCAGCTTGTTCCG | 60 | 55 |  |
| 23 | SbSMP-2 | F:GCATCGTCACCGAGTTCGT | 60 | 57 | 131 |
|  |  | R: ATCCTTACCGACCCCACCA | 60 | 57 |  |
| 24 | SbACP2 | F:ACGAACTTGTTGCGGCAGAAG | 58.5 | 52.4 | 110 |
|  |  | R: GAACAAGAAGGGATGCGCTGG | 58.8 | 57.1 |  |
| 25 | SbEF-P | F:TGAAGCGGGTGAGAAGATTGT | 56.5 | 47.6 | 114 |
|  |  | R: AGCCAAATCATACTCGCCCA | 56.7 | 50 |  |
