# Supplementary material for: Genome-scale identification, classification, and tissue specific expression analysis of late embryogenesis abundant (LEA) genes under abiotic stress conditions in Sorghum bicolor L
Source: PLoS One. 2019 Jan 16;14(1):e0209980. doi: 10.1371/journal.pone.0209980 (PMC6335061; doi:10.1371/journal.pone.0209980)
Supplement: S2 Table — (DOCX) [file pone.0209980.s005.docx]

| Name  **S2 Table.** Types of protein kinases in the phosphorylation of SbLEAs | Phosphorylation sites | | | PKC | CK2 | RSK | PKA | UNSP | EGFR | INSR | PKG | CK1 | DNAPK | CdC2 | p38MAPK | CDK5 | GSK3 | ATM |
| --- | --- | --- | --- | --- | --- | --- | --- | --- | --- | --- | --- | --- | --- | --- | --- | --- | --- | --- |
|  | Serine | Threonine | Tyrosine |  |  |  |  |  |  |  |  |  |  |  |  |  |  |  |
| Sb02g018540 | 22 | 17 | 3 | 18 | 0 | 0 | 6 | 18 | 1 | 0 | 1 | 0 | 3 | 4 | 3 | 3 | 1 | 1 |
| Sb06g026900 | 3 | 6 | 1 | 7 | 0 | 0 | 0 | 7 | 0 | 0 | 0 | 0 | 0 | 0 | 0 | 0 | 0 | 0 |
| Sb07g007690 | 5 | 8 | 1 | 8 | 0 | 1 | 1 | 9 | 0 | 0 | 0 | 0 | 2 | 1 | 1 | 1 | 0 | 1 |
| Sb10g000930 | 10 | 10 | 0 | 11 | 3 | 1 | 3 | 11 | 0 | 0 | 2 | 0 | 2 | 2 | 0 | 0 | 0 | 0 |
| Sb10g012265 | 22 | 14 | 3 | 21 | 2 | 1 | 6 | 22 | 1 | 0 | 3 | 0 | 1 | 4 | 1 | 0 | 0 | 0 |
| Sb01g000200 | 9 | 5 | 4 | 4 | 0 | 0 | 3 | 9 | 1 | 1 | 2 | 0 | 0 | 1 | 0 | 1 | 1 | 0 |
| Sb01g001660 | 11 | 12 | 2 | 14 | 2 | 0 | 1 | 15 | 0 | 1 | 1 | 2 | 1 | 2 | 0 | 0 | 0 | 0 |
| Sb01g002130 | 8 | 7 | 1 | 7 | 0 | 0 | 3 | 7 | 0 | 1 | 1 | 2 | 0 | 1 | 0 | 0 | 1 | 0 |
| Sb01g011230 | 22 | 10 | 6 | 12 | 1 | 3 | 6 | 26 | 1 | 1 | 1 | 0 | 1 | 3 | 3 | 3 | 2 | 2 |
| Sb01g016860 | 7 | 5 | 1 | 2 | 0 | 0 | 1 | 9 | 0 | 0 | 1 | 1 | 2 | 3 | 0 | 1 | 1 | 0 |
| Sb01g018550 | 22 | 17 | 2 | 22 | 1 | 0 | 7 | 24 | 0 | 1 | 2 | 0 | 1 | 7 | 1 | 1 | 1 | 1 |
| Sb01g030000 | 6 | 5 | 0 | 4 | 0 | 0 | 1 | 5 | 0 | 0 | 2 | 0 | 0 | 1 | 1 | 1 | 1 | 0 |
| Sb01g040310 | 13 | 3 | 3 | 6 | 1 | 0 | 1 | 13 | 0 | 0 | 0 | 1 | 1 | 2 | 2 | 3 | 2 | 0 |
| Sb02g006180 | 57 | 17 | 7 | 22 | 9 | 5 | 13 | 41 | 0 | 2 | 3 | 4 | 3 | 17 | 3 | 3 | 3 | 0 |
| Sb02g008820 | 17 | 13 | 2 | 13 | 5 | 0 | 2 | 16 | 0 | 0 | 1 | 2 | 0 | 2 | 1 | 0 | 0 | 0 |
| Sb02g002730 | 42 | 11 | 2 | 29 | 1 | 2 | 9 | 38 | 0 | 1 | 1 | 3 | 1 | 15 | 0 | 2 | 0 | 1 |
| Sb02g017900 | 14 | 6 | 3 | 7 | 1 | 0 | 4 | 13 | 0 | 0 | 2 | 0 | 0 | 2 | 1 | 1 | 1 | 1 |
| Sb02g025570 | 20 | 7 | 1 | 9 | 0 | 0 | 4 | 14 | 1 | 0 | 0 | 1 | 0 | 6 | 1 | 2 | 0 | 0 |
| Sb02g030840 | 16 | 11 | 0 | 10 | 0 | 0 | 5 | 20 | 0 | 0 | 1 | 1 | 0 | 1 | 2 | 0 | 0 | 0 |
| Sb02g035010 | 4 | 9 | 2 | 5 | 0 | 0 | 1 | 9 | 0 | 0 | 1 | 0 | 1 | 2 | 1 | 1 | 1 | 0 |
| Sb02g035250 | 6 | 5 | 1 | 7 | 0 | 0 | 3 | 5 | 0 | 0 | 1 | 0 | 1 | 1 | 1 | 0 | 0 | 1 |
| Sb02g038356 | 35 | 8 | 2 | 22 | 0 | 5 | 9 | 35 | 0 | 1 | 1 | 3 | 1 | 13 | 0 | 2 | 0 | 1 |
| Sb03g001170 | 6 | 4 | 2 | 4 | 1 | 0 | 1 | 6 | 0 | 2 | 0 | 0 | 1 | 3 | 0 | 0 | 0 | 0 |
| Sb03g025840 | 17 | 9 | 0 | 10 | 2 | 4 | 3 | 20 | 0 | 0 | 1 | 3 | 0 | 6 | 2 | 1 | 1 | 0 |
| Sb03g033900 | 9 | 11 | 3 | 8 | 5 | 1 | 1 | 11 | 1 | 2 | 2 | 2 | 1 | 1 | 1 | 1 | 0 | 0 |
| Sb04g009840 | 34 | 15 | 1 | 19 | 2 | 3 | 5 | 33 | 0 | 1 | 6 | 2 | 1 | 11 | 3 | 4 | 2 | 1 |
| Sb04g022010 | 15 | 7 | 0 | 8 | 0 | 0 | 4 | 10 | 0 | 0 | 1 | 1 | 2 | 6 | 2 | 0 | 1 | 1 |
| Sb04g023155 | 41 | 14 | 1 | 19 | 2 | 0 | 10 | 26 | 0 | 1 | 1 | 1 | 2 | 16 | 2 | 1 | 1 | 1 |
| Sb04g032250 | 13 | 4 | 0 | 4 | 7 | 1 | 2 | 12 | 0 | 0 | 0 | 3 | 1 | 2 | 1 | 0 | 0 | 0 |
| Sb04g032400 | 6 | 10 | 1 | 10 | 0 | 1 | 3 | 12 | 0 | 0 | 0 | 1 | 0 | 0 | 1 | 1 | 1 | 0 |
| Sb05g001340 | 28 | 17 | 3 | 16 | 2 | 5 | 10 | 28 | 0 | 0 | 2 | 1 | 3 | 5 | 1 | 4 | 3 | 1 |
| Sb05g003630 | 28 | 12 | 0 | 15 | 0 | 4 | 5 | 23 | 0 | 0 | 1 | 0 | 2 | 13 | 1 | 4 | 1 | 0 |
| Sb05g003631 | 9 | 9 | 3 | 8 | 0 | 0 | 5 | 9 | 1 | 0 | 1 | 1 | 0 | 2 | 1 | 0 | 1 | 0 |
| Sb06g016230 | 11 | 5 | 0 | 7 | 1 | 0 | 4 | 7 | 0 | 0 | 0 | 0 | 0 | 1 | 1 | 1 | 1 | 0 |
| Sb06g029380 | 13 | 6 | 1 | 7 | 0 | 0 | 2 | 15 | 1 | 0 | 1 | 3 | 0 | 4 | 0 | 1 | 0 | 0 |
| Sb06g032920 | 8 | 7 | 2 | 7 | 1 | 1 | 2 | 9 | 1 | 0 | 3 | 0 | 0 | 1 | 1 | 1 | 3 | 0 |
| Sb06g033570 | 11 | 10 | 3 | 12 | 0 | 1 | 3 | 10 | 1 | 0 | 1 | 0 | 2 | 2 | 1 | 0 | 0 | 0 |
| Sb06g033580 | 5 | 7 | 3 | 6 | 0 | 0 | 3 | 4 | 1 | 1 | 0 | 0 | 0 | 2 | 1 | 2 | 1 | 0 |
| Sb07g000360 | 9 | 3 | 0 | 7 | 0 | 0 | 2 | 5 | 0 | 0 | 0 | 0 | 0 | 1 | 1 | 1 | 0 | 0 |
| Sb08g001610 | 18 | 6 | 0 | 5 | 0 | 0 | 1 | 17 | 0 | 0 | 0 | 1 | 0 | 4 | 3 | 6 | 6 | 0 |
| Sb08g003690 | 20 | 7 | 1 | 8 | 1 | 1 | 3 | 16 | 0 | 0 | 2 | 2 | 0 | 8 | 3 | 2 | 1 | 0 |
| Sb08g003720 | 26 | 18 | 4 | 15 | 2 | 3 | 11 | 33 | 1 | 0 | 4 | 2 | 0 | 9 | 3 | 5 | 5 | 0 |
| Sb09g023690 | 11 | 7 | 2 | 9 | 0 | 0 | 3 | 9 | 0 | 0 | 1 | 2 | 1 | 1 | 0 | 1 | 1 | 0 |
| Sb09g026230 | 9 | 14 | 5 | 11 | 4 | 0 | 1 | 17 | 0 | 2 | 2 | 0 | 0 | 2 | 2 | 1 | 0 | 0 |
| Sb09g029870 | 11 | 5 | 1 | 4 | 0 | 0 | 5 | 12 | 1 | 0 | 2 | 0 | 0 | 2 | 1 | 0 | 1 | 0 |
| Sb01g033070 | 5 | 3 | 0 | 5 | 0 | 0 | 1 | 2 | 0 | 0 | 1 | 0 | 0 | 0 | 1 | 0 | 0 | 0 |
| Sb03g009860 | 7 | 1 | 0 | 4 | 0 | 0 | 0 | 5 | 0 | 0 | 0 | 0 | 0 | 3 | 0 | 0 | 0 | 0 |
| Sb03g012940 | 8 | 4 | 2 | 5 | 1 | 0 | 2 | 9 | 2 | 0 | 0 | 0 | 0 | 1 | 1 | 2 | 2 | 0 |
| Sb03g012950 | 0 | 2 | 1 | 2 | 0 | 0 | 0 | 2 | 1 | 0 | 0 | 0 | 0 | 0 | 0 | 0 | 0 | 0 |
| Sb04g023310 | 4 | 1 | 0 | 4 | 1 | 0 | 0 | 3 | 0 | 0 | 0 | 1 | 0 | 0 | 0 | 0 | 0 | 0 |
| Sb07g022150 | 3 | 2 | 2 | 4 | 0 | 0 | 1 | 4 | 1 | 0 | 0 | 0 | 0 | 0 | 0 | 0 | 0 | 0 |
| Sb09g018000 | 3 | 7 | 0 | 5 | 3 | 0 | 0 | 6 | 0 | 0 | 0 | 0 | 0 | 0 | 0 | 0 | 0 | 0 |
| Sb01g036790 | 12 | 19 | 4 | 7 | 4 | 1 | 2 | 22 | 0 | 0 | 4 | 3 | 1 | 3 | 0 | 0 | 0 | 0 |
| Sb01g046000 | 19 | 12 | 4 | 10 | 7 | 1 | 4 | 30 | 0 | 2 | 1 | 3 | 0 | 3 | 0 | 0 | 0 | 0 |
| Sb03g032380 | 8 | 10 | 0 | 9 | 2 | 0 | 2 | 12 | 0 | 0 | 0 | 0 | 0 | 0 | 0 | 0 | 0 | 0 |
| Sb06g028110 | 19 | 22 | 3 | 13 | 3 | 0 | 3 | 26 | 0 | 1 | 3 | 3 | 1 | 5 | 2 | 2 | 0 | 1 |
| Sb09g027110 | 14 | 13 | 1 | 15 | 0 | 0 | 3 | 20 | 0 | 0 | 3 | 1 | 1 | 1 | 0 | 0 | 0 | 1 |
| Sb09g016830 | 17 | 11 | 5 | 8 | 6 | 2 | 4 | 26 | 3 | 0 | 3 | 1 | 1 | 3 | 0 | 0 | 0 | 0 |
| Sb02g028010 | 2 | 4 | 2 | 1 | 1 | 0 | 1 | 5 | 0 | 1 | 1 | 0 | 0 | 1 | 0 | 0 | 0 | 0 |
| Sb01g008210 | 7 | 11 | 1 | 6 | 1 | 0 | 1 | 10 | 0 | 0 | 2 | 0 | 1 | 1 | 1 | 2 | 1 | 1 |
| Sb01g046490 | 8 | 7 | 0 | 4 | 3 | 1 | 3 | 9 | 0 | 0 | 2 | 1 | 1 | 3 | 0 | 0 | 0 | 1 |
| Sb07g015410 | 2 | 2 | 1 | 0 | 0 | 0 | 0 | 3 | 3 | 0 | 1 | 0 | 0 | 1 | 0 | 1 | 0 | 0 |
| [Sb03g027020](http://ensembl.gramene.org/Sorghum_bicolor/Gene/Summary?db=core;g=SORBI_003G270200;tl=otuXOSFsJhvJF8vM-7798-3507751) | 21 | 10 | 7 | 14 | 4 | 1 | 3 | 32 | 2 | 2 | 2 | 1 | 0 | 6 | 1 | 1 | 0 | 0 |
| Sb03g032255 | 16 | 4 | 6 | 10 | 1 | 1 | 1 | 20 | 2 | 2 | 0 | 2 | 0 | 7 | 0 | 0 | 0 | 0 |
| Sb03g037700 | 27 | 4 | 2 | 13 | 1 | 2 | 3 | 28 | 0 | 0 | 0 | 1 | 1 | 11 | 1 | 3 | 1 | 0 |
| Sb09g018420 | 8 | 5 | 2 | 6 | 2 | 1 | 0 | 12 | 0 | 2 | 0 | 4 | 0 | 4 | 0 | 0 | 0 | 0 |
| Sb09g029860 | 18 | 13 | 2 | 14 | 1 | 0 | 4 | 19 | 0 | 1 | 1 | 1 | 0 | 6 | 0 | 0 | 0 | 0 |
| Sb10g003700 | 13 | 28 | 9 | 20 | 4 | 1 | 1 | 25 | 5 | 3 | 0 | 15 | 2 | 9 | 0 | 0 | 0 | 0 |

(PKC: Protein Kinase C, CK2: Casein Kinase 2, RSK1: Ribosomal S6 Kinase 1, PKA: Protein Kinase A, CK1: Casein Kinase, DNAPK: DNA dependant Protein Kinase, ATM: Ataxia-telangiectasia mutated **kinase**, EGFR: epidermal growth factor receptor, INSR: insulin receptor tyrosine **kinase, PKG:** Protein **Kinase** G, CDK: Cyclin dependent kinases)
