# Supplementary material for: Genome-scale identification, classification, and tissue specific expression analysis of late embryogenesis abundant (LEA) genes under abiotic stress conditions in Sorghum bicolor L
Source: PLoS One. 2019 Jan 16;14(1):e0209980. doi: 10.1371/journal.pone.0209980 (PMC6335061; doi:10.1371/journal.pone.0209980)
Supplement: S3 Table — (DOCX) [file pone.0209980.s006.docx]

| miRNA_Acc.  **S3 Table.** miRNAs targets SbLEA genes | Target_Acc. | Expectation | UPE$ | miRNA_start | miRNA_end | Target_start | Target_end | miRNA_aligned_fragment | Target_aligned_fragment | Inhibition target | Multiplicity |
| --- | --- | --- | --- | --- | --- | --- | --- | --- | --- | --- | --- |
| sbi-miR437x-5p | Sb01g000200 | 0 | -1 | 1 | 24 | 1735 | 1758 | UAGAGUUGUCCUAAGUCAAACUUU | AAAGUUUGACUUAGGACAACUCUA | Cleavage | 1 |
| sbi-miR437x-5p | Sb09g026230 | 0 | -1 | 1 | 24 | 777 | 800 | UAGAGUUGUCCUAAGUCAAACUUU | AAAGUUUGACUUAGGACAACUCUA | Cleavage | 1 |
| sbi-miR6225-5p | Sb08g003690 | 0 | -1 | 1 | 24 | 3210 | 3233 | AACUAGACUCAAAAGAUUCAUCUC | GAGCUGAAUCUUUUGAGUCUAGUU | Cleavage | 3 |
| sbi-miR437x-5p | Sb01g002130 | 1 | -1 | 1 | 24 | 673 | 696 | UAGAGUUGUCCUAAGUCAAACUUU | AAAGUUUAACUUAGGACAACUCUA | Cleavage | 1 |
| sbi-miR6225-5p | Sb09g018000 | 1 | -1 | 1 | 24 | 1918 | 1941 | AACUAGACUCAAAAGAUUCAUCUC | GAGACAAAUCUUUUGAGUCUAGUU | Cleavage | 1 |
| sbi-miR6235-5p | Sb03g001170 | 1 | -1 | 1 | 24 | 3448 | 3471 | UUGUGAGAGAAAAAUACUGUUGGC | UGUAACAGUAGUUUUCUCUCACAA | Cleavage | 1 |
| sbi-miR437x-3p | Sb01g002130 | 1.5 | -1 | 1 | 24 | 494 | 517 | AUUUGACUGACACGGAUUCUAGGA | UUCUAGAAUCCGUGCCAGUCAAAU | Translation | 1 |
| sbi-miR5389 | Sb09g029870 | 1.5 | -1 | 1 | 21 | 3652 | 3672 | GCUUGAGUUUAUCAGCCGAGU | AUUCGGCUGAUAAACUCAAAC | Cleavage | 1 |
| sbi-miR5389 | Sb09g029860 | 1.5 | -1 | 1 | 21 | 3652 | 3672 | GCUUGAGUUUAUCAGCCGAGU | AUUCGGCUGAUAAACUCAAAC | Cleavage | 1 |
| sbi-miR6220-5p | Sb04g032400 | 1.5 | -1 | 1 | 24 | 3027 | 3050 | CUCCAUCCUAAAUUAUAAGACAUU | AAUGUCUUAUAAUUUGGGAUGGAG | Cleavage | 1 |
| sbi-miR6225-3p | Sb03g001170 | 1.5 | -1 | 1 | 24 | 4534 | 4557 | GAAACGAAUCUUUUAAGUCUAAUU | AAUUAGACUUAAAAGAUUCGUCUC | Cleavage | 1 |
| sbi-miR6225-5p | Sb08g003690 | 1.5 | -1 | 1 | 24 | 2931 | 2954 | AACUAGACUCAAAAGAUUCAUCUC | GAGACGAAUCUUUUGAGCCUAGUU | Cleavage | 3 |
| sbi-miR6225-5p | Sb06g029380 | 1.5 | -1 | 1 | 24 | 1277 | 1300 | AACUAGACUCAAAAGAUUCAUCUC | GAGAUGAAUCUUUUGAGCCUAGUU | Cleavage | 1 |
| sbi-miR6225-5p | Sb01g040310 | 1.5 | -1 | 1 | 24 | 927 | 950 | AACUAGACUCAAAAGAUUCAUCUC | GAGAUGAAUCUUUUGAGCCUAGUU | Cleavage | 1 |
| sbi-miR6225-5p | Sb08g001610 | 1.5 | -1 | 1 | 24 | 2410 | 2433 | AACUAGACUCAAAAGAUUCAUCUC | GAGAUGAAUCUUUUAAGUCUAGUU | Translation | 1 |
| sbi-miR6225-5p | Sb03g009860 | 1.5 | -1 | 1 | 24 | 2679 | 2702 | AACUAGACUCAAAAGAUUCAUCUC | GAGACGAAUCUUUUGAGCCUAGUU | Cleavage | 1 |
| sbi-miR6225-5p | Sb07g022150 | 1.5 | -1 | 1 | 24 | 3137 | 3160 | AACUAGACUCAAAAGAUUCAUCUC | GAGACGAAUCUUUUGAGCCUAGUU | Cleavage | 1 |
| sbi-miR6225-5p | Sb06g028110 | 1.5 | -1 | 1 | 24 | 8625 | 8648 | AACUAGACUCAAAAGAUUCAUCUC | GAGACGAAUCUUUUGAUUCUAGUU | Cleavage | 1 |
| sbi-miR6235-5p | Sb05g003630 | 1.5 | -1 | 1 | 24 | 3708 | 3731 | UUGUGAGAGAAAAAUACUGUUGGC | UUCAGCAGUAUUUUUCUCUCAUAA | Cleavage | 1 |
| sbi-miR5568f-3p | Sb08g001610 | 2 | -1 | 1 | 21 | 9465 | 9485 | GUCUUAUAAUUUGGAAUGGAG | CUCAAUUUCAAAUUAUAAGAC | Cleavage | 1 |
| sbi-miR6225-3p | Sb08g001610 | 2 | -1 | 1 | 24 | 7078 | 7101 | GAAACGAAUCUUUUAAGUCUAAUU | AAUUAGAUUUAAAAGAUUCGUUUU | Cleavage | 1 |
| sbi-miR5567 | Sb09g023690 | 2.5 | -1 | 1 | 24 | 3008 | 3032 | UUAAUGAUUCAUGUAUGUGUC-CAA | UUGCGGCACAUGCAUGAAGCAUUAA Cleavage | | 1 |
| sbi-miR5567 | Sb02g030840 | 2.5 | -1 | 1 | 24 | 7697 | 7721 | UUAAUGAUUCAUGUAUGUGUC-CAA | UUGCGGCACAUGCAUGAAGCAUUAA Cleavage | | 1 |
| sbi-miR5568c-5p | Sb01g016860 | 2.5 | -1 | 1 | 21 | 512 | 532 | UCUGUUCCAAAUUGUAAGUCG | UGACUUAUAAUUUGGAACGGA | Cleavage | 1 |
| sbi-miR5568c-5p | Sb03g012940 | 2.5 | -1 | 1 | 21 | 4621 | 4641 | UCUGUUCCAAAUUGUAAGUCG | CGACUUAUAAUUUAGAACAGA | Cleavage | 1 |
| sbi-miR5568c-5p | Sb06g028110 | 2.5 | -1 | 1 | 21 | 6243 | 6263 | UCUGUUCCAAAUUGUAAGUCG | UGACUUAUAAUUUGGAACCGA | Cleavage | 1 |
| sbi-miR5568f-5p | Sb04g032400 | 2.5 | -1 | 1 | 21 | 3029 | 3049 | UCCAUUCCAAAUUGUAAGAUG | UGUCUUAUAAUUUGGGAUGGA | Cleavage | 1 |
| sbi-miR6218-5p | Sb07g015410 | 2.5 | -1 | 1 | 21 | 1003 | 1023 | CGAAAAUCACGAAACUUGUCG | CGACAAGUUUCGUGAUUUUUC | Cleavage | 1 |
| sbi-miR6220-3p | Sb02g035010 | 2.5 | -1 | 1 | 24 | 2992 | 3015 | AUGCCUUAUAAUUUGGGAUGGAGA | CCUUUAUUCCAAAUUAUAAGGUAU | Cleavage | 2 |
| sbi-miR6220-3p | Sb02g035010 | 2.5 | -1 | 1 | 24 | 334 | 357 | AUGCCUUAUAAUUUGGGAUGGAGA | UUUUCAUCUCAAAUUAUAAGUCAU | Cleavage | 2 |
| sbi-miR6220-5p | Sb06g033570 | 2.5 | -1 | 1 | 24 | 4122 | 4145 | CUCCAUCCUAAAUUAUAAGACAUU | AAUGACUUACAAUUUGGGAUGGAG | Cleavage | 1 |
| sbi-miR6225-3p | Sb04g023310 | 2.5 | -1 | 1 | 24 | 6311 | 6334 | GAAACGAAUCUUUUAAGUCUAAUU | AACUAGGCUUAAAAGAUUCGUCUC | Cleavage | 2 |
| sbi-miR6225-3p | Sb04g023310 | 2.5 | -1 | 1 | 24 | 8313 | 8336 | GAAACGAAUCUUUUAAGUCUAAUU | AACUAGACUCAAAAGAUUCGUCUC | Cleavage | 2 |
| sbi-miR6225-3p | Sb04g023310 | 2.5 | -1 | 1 | 24 | 6311 | 6334 | GAAACGAAUCUUUUAAGUCUAAUU | AACUAGGCUUAAAAGAUUCGUCUC | Cleavage | 2 |
| sbi-miR6225-3p | Sb04g023310 | 2.5 | -1 | 1 | 24 | 8313 | 8336 | GAAACGAAUCUUUUAAGUCUAAUU | AACUAGACUCAAAAGAUUCGUCUC | Cleavage | 2 |
| sbi-miR6225-3p | Sb04g032250 | 2.5 | -1 | 1 | 24 | 1259 | 1282 | GAAACGAAUCUUUUAAGUCUAAUU | AACUAGGCUUAAAAGAUUCGUCUC | Cleavage | 1 |
| sbi-miR6225-3p | Sb08g003720 | 2.5 | -1 | 1 | 24 | 3225 | 3248 | GAAACGAAUCUUUUAAGUCUAAUU | AACUAGGCUUAAAAGAUUCGUCUC | Cleavage | 1 |
| sbi-miR6225-3p | Sb06g032920 | 2.5 | -1 | 1 | 24 | 3539 | 3562 | GAAACGAAUCUUUUAAGUCUAAUU | UCUUAGGCUUAAAAGAUUCGUCUC | Cleavage | 1 |
| sbi-miR6225-3p | Sb06g028110 | 2.5 | -1 | 1 | 24 | 2300 | 2323 | GAAACGAAUCUUUUAAGUCUAAUU | AACUAGACUCAAAAGAUUCGUCUC | Cleavage | 1 |
| sbi-miR6225-3p | Sb05g003630 | 2.5 | -1 | 1 | 24 | 786 | 809 | GAAACGAAUCUUUUAAGUCUAAUU | AACUAGUCUUAAAAGAUUCGUCUC | Cleavage | 1 |
| sbi-miR6225-5p | Sb08g003690 | 2.5 | -1 | 1 | 24 | 1506 | 1529 | AACUAGACUCAAAAGAUUCAUCUC | GAGACAAAUCUUUUGAGCCUAGUU | Cleavage | 3 |
| sbi-miR6225-5p | Sb02g030840 | 2.5 | -1 | 1 | 24 | 7336 | 7359 | AACUAGACUCAAAAGAUUCAUCUC | GAGAUGAAUAUUUUGAGCCUAGUU | Cleavage | 1 |
| sbi-miR6225-5p | Sb07g015410 | 2.5 | -1 | 1 | 24 | 3633 | 3656 | AACUAGACUCAAAAGAUUCAUCUC | GCGACGAAUUUUUUAAGUCUAGUU | Translation | 1 |
| sbi-miR437x-3p | Sb01g000200 | 3 | -1 | 1 | 24 | 1557 | 1580 | AUUUGACUGACACGGAUUCUAGGA | UUCUAGGAUUCGUGUCAGUCAAAC | Cleavage | 1 |
| sbi-miR5568g-5p | Sb04g023310 | 3 | -1 | 1 | 21 | 3297 | 3317 | CAAAUUAUAAGAUGUUUUGGC | AUAAAAAUAUCUUAUAAUUUA | Cleavage | 1 |
| sbi-miR5568g-5p | Sb04g023310 | 3 | -1 | 1 | 21 | 3297 | 3317 | CAAAUUAUAAGAUGUUUUGGC | AUAAAAAUAUCUUAUAAUUUA | Cleavage | 1 |
