# Supplementary material for: Genome-scale identification, classification, and tissue specific expression analysis of late embryogenesis abundant (LEA) genes under abiotic stress conditions in Sorghum bicolor L
Source: PLoS One. 2019 Jan 16;14(1):e0209980. doi: 10.1371/journal.pone.0209980 (PMC6335061; doi:10.1371/journal.pone.0209980)
Supplement: S4 Table — (DOCX) [file pone.0209980.s007.docx]

| **Stress** | **Abiotic stress** | | | | | | | | | | | | | **Hormone** | | | | | | | | | **Developmental** | | | | | **Biotic** | | | | | **others** | | | |
| --- | --- | --- | --- | --- | --- | --- | --- | --- | --- | --- | --- | --- | --- | --- | --- | --- | --- | --- | --- | --- | --- | --- | --- | --- | --- | --- | --- | --- | --- | --- | --- | --- | --- | --- | --- | --- |
| **Gene** | **DRE** | **DPBF** | **MYB** | **MYC** | **HSE** | **LTRE** | **GT1GM** | **SP1** | **G-Box** | **Cu-res** | **CBFHV** | **I-Box** | **Anaero** | **ABRE** | **TCA** | **TGACG** | **CGTCA** | **GARE** | **TGA** | **ERE** | **P-Box** | **Motif IIb** | **SKn** | **Pollen** | **CCGTCC** | **CIRCADI** | **GCN4** | **WBOXATNPR1** | **WBOXNTCH** | **WBOXNTERF3** | **TC-RICH** | **WBBOXPCWRKY1** | **TKST1** | **CGCG** | **O2** | **AT rich** |
| Sb02g018540 | 2 | 2 | 7 | 14 | 1 | 2 | 5 | 0 | 1 | 6 | 1 | 3 | 0 | 1 | 6 | 5 | 2 | 1 | 4 | 6 | 1 | 2 | 6 | 7 | 3 | 1 | 2 | 3 | 5 | 9 | 6 | 1 | 7 | 1 | 5 | 4 |
| Sb06g026900 | 7 | 4 | 15 | 20 | 1 | 6 | 5 | 7 | 5 | 14 | 7 | 1 | 4 | 19 | 14 | 8 | 4 | 0 | 2 | 3 | 1 | 1 | 1 | 2 | 2 | 3 | 4 | 5 | 2 | 8 | 1 | 0 | 1 | 16 | 1 | 1 |
| Sb07g007690 | 2 | 3 | 18 | 32 | 1 | 1 | 6 | 2 | 8 | 10 | 2 | 6 | 6 | 16 | 11 | 3 | 1 | 3 | 1 | 1 | 1 | 1 | 5 | 2 | 3 | 0 | 1 | 3 | 2 | 5 | 0 | 0 | 0 | 18 | 2 | 2 |
| Sb10g000930 | 5 | 6 | 13 | 22 | 4 | 7 | 10 | 6 | 6 | 16 | 3 | 7 | 8 | 25 | 10 | 2 | 2 | 0 | 1 | 1 | 1 | 3 | 1 | 6 | 2 | 1 | 3 | 1 | 1 | 2 | 1 | 0 | 1 | 5 | 3 | 0 |
| Sb10g012265 | 1 | 2 | 24 | 16 | 0 | 1 | 15 | 2 | 20 | 18 | 1 | 8 | 1 | 13 | 6 | 2 | 1 | 1 | 1 | 2 | 2 | 0 | 2 | 8 | 2 | 3 | 4 | 1 | 0 | 1 | 1 | 0 | 4 | 9 | 0 | 1 |
| Sb01g000200 | 9 | 1 | 20 | 14 | 3 | 7 | 21 | 1 | 12 | 22 | 6 | 4 | 3 | 3 | 5 | 4 | 1 | 0 | 2 | 2 | 1 | 1 | 2 | 7 | 1 | 0 | 1 | 5 | 1 | 4 | 2 | 0 | 2 | 10 | 1 | 0 |
| Sb01g001660 | 0 | 2 | 20 | 26 | 1 | 2 | 8 | 2 | 0 | 12 | 1 | 2 | 2 | 0 | 9 | 2 | 2 | 3 | 2 | 1 | 1 | 0 | 8 | 14 | 3 | 1 | 1 | 7 | 3 | 13 | 1 | 2 | 6 | 3 | 2 | 2 |
| Sb01g002130 | 0 | 2 | 20 | 24 | 4 | 1 | 23 | 3 | 4 | 14 | 0 | 1 | 2 | 4 | 5 | 2 | 3 | 0 | 1 | 5 | 1 | 1 | 5 | 12 | 1 | 0 | 1 | 10 | 1 | 12 | 2 | 3 | 3 | 2 | 0 | 0 |
| Sb01g011230 | 2 | 4 | 17 | 16 | 2 | 1 | 10 | 0 | 3 | 2 | 1 | 1 | 2 | 2 | 4 | 1 | 1 | 1 | 3 | 1 | 2 | 0 | 4 | 10 | 1 | 1 | 0 | 2 | 0 | 8 | 2 | 1 | 7 | 3 | 1 | 1 |
| Sb01g016860 | 2 | 2 | 10 | 4 | 1 | 3 | 7 | 0 | 11 | 4 | 1 | 2 | 1 | 5 | 3 | 2 | 2 | 2 | 2 | 2 | 1 | 0 | 1 | 1 | 3 | 2 | 1 | 0 | 1 | 1 | 1 | 0 | 0 | 5 | 1 | 1 |
| Sb01g018550 | 0 | 3 | 13 | 12 | 2 | 0 | 21 | 1 | 0 | 2 | 0 | 0 | 2 | 3 | 7 | 2 | 0 | 2 | 3 | 4 | 1 | 0 | 2 | 12 | 0 | 2 | 1 | 14 | 2 | 11 | 1 | 3 | 1 | 2 | 1 | 2 |
| Sb01g030000 | 3 | 2 | 2 | 12 | 2 | 3 | 13 | 1 | 1 | 20 | 2 | 6 | 2 | 6 | 10 | 1 | 3 | 3 | 1 | 0 | 1 | 1 | 1 | 13 | 3 | 2 | 2 | 2 | 0 | 4 | 1 | 0 | 14 | 0 | 3 | 1 |
| Sb01g040310 | 4 | 2 | 14 | 16 | 3 | 3 | 12 | 4 | 6 | 2 | 6 | 1 | 3 | 6 | 9 | 3 | 1 | 0 | 1 | 1 | 1 | 3 | 5 | 3 | 2 | 3 | 0 | 3 | 0 | 5 | 1 | 0 | 0 | 7 | 1 | 1 |
| Sb02g006180 | 0 | 5 | 9 | 34 | 1 | 2 | 20 | 1 | 1 | 6 | 0 | 9 | 5 | 2 | 7 | 1 | 1 | 0 | 1 | 2 | 3 | 0 | 5 | 6 | 1 | 0 | 1 | 3 | 1 | 7 | 2 | 0 | 3 | 1 | 1 | 1 |
| Sb02g008820 | 0 | 0 | 12 | 14 | 3 | 0 | 23 | 1 | 1 | 6 | 0 | 8 | 2 | 1 | 4 | 3 | 3 | 3 | 1 | 1 | 1 | 1 | 3 | 15 | 1 | 2 | 4 | 1 | 0 | 4 | 1 | 0 | 10 | 2 | 4 | 3 |
| Sb02g002730 | 1 | 2 | 9 | 4 | 3 | 2 | 15 | 2 | 0 | 0  **S4 Table** . Conserved *cis*-acting elements in *LEA* promoters of *Sorghum* | 1 | 1 | 1 | 0 | 2 | 1 | 0 | 0 | 1 | 1 | 1 | 0 | 3 | 6 | 0 | 1 | 3 | 1 | 0 | 3 | 2 | 0 | 6 | 2 | 0 | 0 |
| Sb02g017900 | 3 | 3 | 18 | 16 | 4 | 4 | 32 | 1 | 0 | 2 | 3 | 7 | 2 | 2 | 12 | 1 | 2 | 1 | 3 | 4 | 5 | 1 | 3 | 17 | 1 | 3 | 2 | 3 | 1 | 6 | 2 | 0 | 5 | 1 | 0 | 1 |
| Sb02g025570 | 13 | 2 | 22 | 10 | 0 | 7 | 8 | 9 | 17 | 8 | 13 | 1 | 4 | 6 | 5 | 1 | 1 | 1 | 2 | 1 | 1 | 3 | 2 | 1 | 1 | 2 | 1 | 1 | 0 | 3 | 0 | 0 | 2 | 13 | 3 | 1 |
| Sb02g030840 | 3 | 2 | 15 | 16 | 3 | 5 | 20 | 3 | 9 | 14 | 5 | 7 | 7 | 8 | 6 | 1 | 7 | 0 | 1 | 1 | 1 | 1 | 1 | 8 | 2 | 0 | 1 | 3 | 2 | 4 | 0 | 0 | 6 | 6 | 0 | 1 |
| Sb02g035010 | 1 | 2 | 19 | 14 | 1 | 7 | 12 | 2 | 10 | 20 | 2 | 1 | 9 | 7 | 8 | 1 | 1 | 1 | 4 | 1 | 1 | 1 | 1 | 6 | 1 | 0 | 1 | 1 | 0 | 3 | 3 | 1 | 4 | 6 | 0 | 0 |
| Sb02g035250 | 0 | 2 | 25 | 16 | 1 | 1 | 17 | 0 | 2 | 2 | 0 | 9 | 2 | 3 | 7 | 3 | 0 | 2 | 1 | 0 | 3 | 0 | 2 | 4 | 0 | 1 | 2 | 2 | 2 | 6 | 1 | 1 | 4 | 0 | 0 | 0 |
| Sb02g038356 | 1 | 4 | 21 | 24 | 2 | 2 | 18 | 4 | 2 | 10 | 1 | 7 | 1 | 1 | 12 | 1 | 1 | 0 | 1 | 2 | 1 | 1 | 5 | 11 | 2 | 1 | 0 | 2 | 3 | 7 | 0 | 0 | 2 | 7 | 1 | 0 |
| Sb03g001170 | 3 | 3 | 23 | 8 | 1 | 4 | 23 | 1 | 9 | 6 | 2 | 7 | 3 | 3 | 6 | 1 | 2 | 2 | 2 | 2 | 1 | 0 | 6 | 5 | 0 | 1 | 0 | 3 | 0 | 4 | 2 | 1 | 5 | 6 | 0 | 0 |
| Sb03g025840 | 0 | 2 | 9 | 4 | 2 | 0 | 9 | 1 | 0 | 2 | 0 | 5 | 8 | 1 | 2 | 2 | 1 | 1 | 0 | 3 | 2 | 0 | 2 | 5 | 0 | 0 | 0 | 2 | 0 | 4 | 0 | 2 | 4 | 1 | 0 | 0 |
| Sb03g033900 | 1 | 1 | 19 | 18 | 4 | 5 | 7 | 3 | 0 | 12 | 0 | 0 | 1 | 1 | 3 | 1 | 1 | 2 | 0 | 2 | 1 | 0 | 4 | 4 | 0 | 1 | 1 | 4 | 2 | 9 | 1 | 1 | 3 | 5 | 2 | 0 |
| Sb04g009840 | 3 | 2 | 14 | 14 | 2 | 3 | 22 | 5 | 0 | 12 | 5 | 2 | 13 | 2 | 2 | 1 | 0 | 0 | 0 | 5 | 2 | 1 | 9 | 2 | 1 | 1 | 1 | 1 | 1 | 1 | 0 | 0 | 2 | 6 | 1 | 1 |
| Sb04g022010 | 1 | 2 | 20 | 16 | 0 | 1 | 7 | 5 | 2 | 6 | 1 | 4 | 4 | 2 | 10 | 2 | 3 | 1 | 0 | 1 | 1 | 0 | 3 | 0 | 2 | 1 | 0 | 1 | 2 | 2 | 0 | 0 | 3 | 10 | 0 | 0 |
| Sb04g023155 | 2 | 5 | 16 | 14 | 0 | 0 | 0 | 2 | 4 | 12 | 5 | 10 | 1 | 6 | 10 | 2 | 0 | 1 | 1 | 0 | 3 | 1 | 8 | 5 | 2 | 3 | 2 | 3 | 1 | 7 | 1 | 0 | 8 | 1 | 0 | 0 |
| Sb04g032250 | 10 | 3 | 12 | 30 | 1 | 13 | 16 | 5 | 3 | 8 | 9 | 5 | 4 | 6 | 9 | 3 | 2 | 0 | 1 | 3 | 1 | 1 | 4 | 3 | 2 | 0 | 0 | 5 | 0 | 4 | 0 | 0 | 3 | 27 | 0 | 0 |
| Sb04g032400 | 4 | 4 | 15 | 12 | 2 | 1 | 15 | 2 | 1 | 2 | 7 | 2 | 2 | 5 | 17 | 1 | 1 | 2 | 5 | 4 | 2 | 0 | 2 | 5 | 0 | 1 | 0 | 8 | 0 | 14 | 1 | 0 | 2 | 9 | 0 | 0 |
| Sb05g001340 | 4 | 4 | 31 | 24 | 1 | 4 | 23 | 1 | 0 | 12 | 3 | 4 | 2 | 1 | 2 | 1 | 3 | 1 | 1 | 0 | 1 | 1 | 3 | 7 | 3 | 0 | 0 | 0 | 4 | 6 | 0 | 0 | 1 | 2 | 0 | 0 |
| Sb05g003630 | 0 | 4 | 35 | 10 | 1 | 2 | 18 | 2 | 16 | 0 | 10 | 2 | 1 | 9 | 1 | 2 | 3 | 3 | 0 | 1 | 2 | 0 | 9 | 9 | 2 | 2 | 1 | 3 | 3 | 5 | 0 | 0 | 1 | 1 | 0 | 0 |
| Sb05g003631 | 0 | 4 | 6 | 12 | 1 | 1 | 13 | 2 | 1 | 10 | 1 | 2 | 11 | 2 | 4 | 4 | 5 | 0 | 1 | 2 | 2 | 0 | 4 | 9 | 1 | 2 | 1 | 2 | 0 | 4 | 1 | 1 | 2 | 3 | 0 | 0 |
| Sb06g016230 | 6 | 2 | 21 | 30 | 2 | 3 | 7 | 3 | 1 | 8 | 6 | 6 | 4 | 3 | 10 | 5 | 2 | 0 | 7 | 0 | 3 | 1 | 10 | 7 | 1 | 1 | 0 | 9 | 0 | 7 | 0 | 1 | 0 | 19 | 0 | 0 |
| Sb06g029380 | 2 | 7 | 19 | 42 | 3 | 5 | 14 | 8 | 4 | 16 | 3 | 0 | 2 | 13 | 1 | 4 | 7 | 0 | 3 | 3 | 1 | 0 | 11 | 7 | 2 | 1 | 0 | 14 | 1 | 6 | 0 | 0 | 2 | 4 | 1 | 0 |
| Sb06g032920 | 7 | 5 | 16 | 14 | 1 | 5 | 11 | 3 | 1 | 14 | 4 | 5 | 2 | 4 | 15 | 11 | 11 | 0 | 8 | 0 | 1 | 1 | 16 | 4 | 5 | 0 | 2 | 14 | 0 | 11 | 0 | 2 | 4 | 8 | 0 | 0 |
| Sb06g033570 | 2 | 1 | 27 | 18 | 2 | 6 | 16 | 8 | 3 | 0 | 4 | 4 | 3 | 20 | 6 | 7 | 5 | 0 | 2 | 1 | 2 | 1 | 17 | 5 | 5 | 2 | 1 | 7 | 4 | 12 | 1 | 5 | 4 | 9 | 0 | 0 |
| Sb06g033580 | 4 | 4 | 20 | 26 | 0 | 2 | 6 | 3 | 5 | 6 | 5 | 2 | 8 | 9 | 12 | 6 | 8 | 1 | 3 | 0 | 1 | 0 | 18 | 8 | 4 | 1 | 2 | 1 | 3 | 8 | 0 | 0 | 5 | 1 | 0 | 0 |
| Sb07g000360 | 0 | 4 | 24 | 22 | 1 | 2 | 12 | 3 | 2 | 8 | 1 | 8 | 6 | 0 | 13 | 6 | 5 | 1 | 0 | 1 | 3 | 20 | 9 | 3 | 0 | 0 | 0 | 4 | 2 | 6 | 0 | 1 | 6 | 5 | 1 | 0 |
| Sb08g001610 | 1 | 1 | 15 | 14 | 2 | 3 | 12 | 4 | 1 | 2 | 1 | 2 | 2 | 3 | 6 | 7 | 2 | 0 | 3 | 2 | 1 | 0 | 4 | 12 | 3 | 0 | 1 | 4 | 2 | 8 | 2 | 0 | 5 | 6 | 1 | 1 |
| Sb08g003690 | 0 | 1 | 14 | 18 | 3 | 1 | 21 | 3 | 1 | 10 | 0 | 7 | 5 | 0 | 13 | 6 | 4 | 0 | 3 | 4 | 2 | 0 | 18 | 5 | 3 | 3 | 5 | 7 | 2 | 9 | 0 | 2 | 5 | 1 | 1 | 0 |
| Sb08g003720 | 1 | 2 | 19 | 18 | 3 | 3 | 17 | 7 | 2 | 14 | 1 | 6 | 5 | 2 | 10 | 7 | 6 | 0 | 1 | 1 | 5 | 0 | 19 | 7 | 2 | 1 | 2 | 2 | 2 | 5 | 0 | 0 | 2 | 0 | 0 | 0 |
| Sb09g023690 | 0 | 9 | 8 | 12 | 1 | 0 | 5 | 5 | 3 | 6 | 0 | 1 | 1 | 14 | 4 | 5 | 4 | 1 | 1 | 0 | 1 | 0 | 3 | 6 | 1 | 0 | 0 | 2 | 0 | 4 | 1 | 0 | 1 | 13 | 0 | 0 |
| Sb09g026230 | 3 | 3 | 22 | 16 | 2 | 3 | 23 | 6 | 2 | 2 | 3 | 6 | 7 | 3 | 11 | 9 | 3 | 0 | 2 | 2 | 2 | 1 | 16 | 6 | 1 | 0 | 2 | 4 | 0 | 7 | 0 | 0 | 1 | 4 | 6 | 1 |
| Sb09g029870 | 7 | 2 | 12 | 12 | 1 | 4 | 7 | 8 | 2 | 6 | 12 | 3 | 6 | 13 | 9 | 3 | 7 | 1 | 3 | 1 | 2 | 0 | 4 | 6 | 0 | 1 | 5 | 2 | 4 | 5 | 1 | 0 | 1 | 17 | 0 | 0 |
| Sb01g033070 | 1 | 6 | 22 | 18 | 5 | 1 | 8 | 1 | 1 | 15 | 2 | 4 | 1 | 9 | 4 | 1 | 2 | 0 | 2 | 1 | 1 | 1 | 6 | 11 | 1 | 0 | 0 | 5 | 1 | 6 | 0 | 1 | 6 | 1 | 4 | 1 |
| Sb03g009860 | 0 | 3 | 12 | 8 | 1 | 1 | 4 | 1 | 1 | 8 | 0 | 9 | 4 | 4 | 2 | 2 | 3 | 2 | 1 | 4 | 2 | 0 | 2 | 10 | 0 | 0 | 0 | 2 | 1 | 5 | 1 | 0 | 6 | 5 | 0 | 1 |
| Sb03g012940 | 2 | 2 | 11 | 7 | 0 | 1 | 2 | 2 | 0 | 7 | 1 | 3 | 4 | 1 | 8 | 4 | 2 | 0 | 1 | 1 | 2 | 2 | 1 | 7 | 1 | 0 | 1 | 1 | 1 | 1 | 1 | 0 | 5 | 10 | 1 | 1 |
| Sb03g012950 | 1 | 1 | 15 | 8 | 2 | 1 | 2 | 6 | 4 | 9 | 2 | 6 | 4 | 4 | 13 | 1 | 7 | 2 | 1 | 1 | 0 | 2 | 4 | 0 | 0 | 0 | 1 | 1 | 4 | 0 | 0 | 0 | 4 | 7 | 0 | 0 |
| Sb04g023310 | 2 | 1 | 14 | 7 | 2 | 3 | 2 | 2 | 2 | 2 | 3 | 2 | 3 | 2 | 8 | 2 | 2 | 2 | 2 | 1 | 1 | 0 | 6 | 5 | 1 | 0 | 0 | 4 | 1 | 5 | 0 | 3 | 1 | 17 | 0 | 0 |
| Sb07g022150 | 4 | 0 | 16 | 5 | 1 | 2 | 1 | 3 | 1 | 5 | 4 | 9 | 2 | 2 | 8 | 3 | 1 | 0 | 5 | 1 | 1 | 0 | 3 | 4 | 1 | 0 | 0 | 5 | 0 | 4 | 1 | 2 | 4 | 9 | 0 | 0 |
| Sb09g018000 | 3 | 4 | 20 | 10 | 1 | 4 | 4 | 1 | 2 | 3 | 2 | 6 | 3 | 3 | 3 | 5 | 3 | 1 | 3 | 1 | 1 | 0 | 4 | 11 | 2 | 0 | 1 | 1 | 1 | 3 | 1 | 1 | 2 | 4 | 1 | 0 |
| Sb01g036790 | 14 | 5 | 13 | 4 | 0 | 9 | 0 | 7 | 3 | 8 | 10 | 4 | 1 | 16 | 20 | 3 | 0 | 1 | 1 | 2 | 1 | 1 | 4 | 1 | 0 | 0 | 0 | 2 | 0 | 4 | 0 | 0 | 0 | 25 | 0 | 0 |
| Sb01g046000 | 5 | 2 | 3 | 2 | 1 | 7 | 2 | 2 | 1 | 8 | 6 | 0 | 1 | 7 | 23 | 2 | 2 | 1 | 0 | 1 | 1 | 1 | 3 | 7 | 2 | 0 | 0 | 2 | 1 | 1 | 1 | 0 | 1 | 17 | 0 | 0 |
| Sb03g032380 | 3 | 5 | 5 | 4 | 3 | 3 | 1 | 1 | 1 | 6 | 1 | 1 | 2 | 12 | 13 | 4 | 1 | 0 | 1 | 2 | 1 | 0 | 3 | 5 | 5 | 0 | 0 | 4 | 1 | 4 | 1 | 0 | 3 | 13 | 0 | 0 |
| Sb06g028110 | 3 | 1 | 5 | 3 | 0 | 4 | 1 | 4 | 2 | 4 | 3 | 1 | 1 | 9 | 16 | 2 | 3 | 0 | 1 | 1 | 0 | 0 | 5 | 2 | 3 | 0 | 1 | 3 | 2 | 5 | 1 | 0 | 1 | 18 | 0 | 0 |
| Sb09g027110 | 2 | 7 | 9 | 9 | 1 | 4 | 2 | 7 | 1 | 3 | 1 | 4 | 1 | 13 | 11 | 0 | 5 | 1 | 2 | 1 | 0 | 0 | 4 | 3 | 2 | 0 | 0 | 1 | 1 | 4 | 1 | 0 | 4 | 5 | 0 | 0 |
| Sb09g016830 | 1 | 4 | 16 | 11 | 0 | 4 | 0 | 1 | 4 | 6 | 4 | 1 | 2 | 12 | 6 | 0 | 3 | 2 | 1 | 0 | 1 | 0 | 1 | 6 | 1 | 0 | 0 | 1 | 1 | 4 | 0 | 0 | 1 | 3 | 0 | 0 |
| Sb02g028010 | 6 | 5 | 14 | 10 | 1 | 8 | 3 | 2 | 3 | 6 | 3 | 3 | 3 | 15 | 7 | 2 | 6 | 2 | 2 | 0 | 0 | 0 | 5 | 10 | 1 | 0 | 1 | 4 | 0 | 2 | 0 | 0 | 7 | 6 | 0 | 0 |
| Sb01g008210 | 8 | 1 | 13 | 9 | 1 | 5 | 1 | 6 | 4 | 6 | 7 | 3 | 4 | 34 | 10 | 2 | 3 | 1 | 1 | 0 | 1 | 3 | 8 | 4 | 2 | 0 | 0 | 3 | 0 | 6 | 0 | 0 | 0 | 18 | 0 | 0 |
| Sb01g046490 | 3 | 2 | 6 | 9 | 2 | 3 | 3 | 5 | 2 | 6 | 2 | 1 | 3 | 21 | 12 | 3 | 2 | 0 | 2 | 0 | 0 | 0 | 4 | 2 | 2 | 0 | 1 | 3 | 0 | 1 | 1 | 1 | 0 | 21 | 0 | 0 |
| Sb07g015410 | 5 | 2 | 18 | 5 | 1 | 3 | 5 | 5 | 2 | 8 | 3 | 10 | 2 | 4 | 3 | 2 | 3 | 0 | 1 | 0 | 1 | 8 | 5 | 3 | 0 | 0 | 0 | 3 | 0 | 2 | 0 | 0 | 3 | 5 | 0 | 0 |
| [Sb03g027020](http://ensembl.gramene.org/Sorghum_bicolor/Gene/Summary?db=core;g=SORBI_003G270200;tl=otuXOSFsJhvJF8vM-7798-3507751) | 7 | 8 | 4 | 3 | 0 | 6 | 2 | 3 | 3 | 10 | 11 | 6 | 2 | 17 | 13 | 3 | 4 | 2 | 0 | 0 | 1 | 0 | 6 | 1 | 2 | 0 | 1 | 1 | 1 | 2 | 1 | 0 | 2 | 10 | 1 | 0 |
| Sb03g032255 | 3 | 3 | 4 | 1 | 0 | 3 | 2 | 0 | 0 | 3 | 3 | 2 | 1 | 6 | 4 | 1 | 3 | 2 | 0 | 0 | 1 | 0 | 4 | 1 | 1 | 0 | 0 | 0 | 1 | 1 | 1 | 0 | 1 | 5 | 0 | 0 |
| Sb03g037700 | 3 | 1 | 11 | 5 | 0 | 4 | 2 | 6 | 4 | 4 | 5 | 11 | 1 | 17 | 5 | 5 | 7 | 0 | 2 | 1 | 1 | 2 | 3 | 2 | 1 | 0 | 0 | 3 | 1 | 5 | 1 | 1 | 3 | 16 | 0 | 0 |
| Sb09g018420 | 8 | 5 | 14 | 9 | 0 | 3 | 0 | 3 | 2 | 14 | 8 | 2 | 0 | 7 | 13 | 2 | 5 | 0 | 1 | 2 | 1 | 0 | 6 | 5 | 0 | 0 | 1 | 0 | 1 | 2 | 0 | 0 | 5 | 3 | 0 | 0 |
| Sb09g029860 | 13 | 5 | 14 | 6 | 0 | 8 | 3 | 13 | 1 | 5 | 10 | 8 | 3 | 8 | 5 | 3 | 3 | 1 | 2 | 0 | 1 | 1 | 7 | 4 | 4 | 0 | 1 | 6 | 2 | 5 | 0 | 2 | 1 | 14 | 0 | 0 |
| Sb10g003700 | 11 | 3 | 20 | 6 | 0 | 6 | 5 | 2 | 5 | 5 | 12 | 3 | 2 | 15 | 10 | 3 | 1 | 2 | 0 | 0 | 2 | 0 | 10 | 11 | 1 | 0 | 0 | 3 | 0 | 3 | 0 | 1 | 3 | 2 | 0 | 0 |

(ABRECTAL: Response to ABA, ANAERO: Anaerobic conditions, ARF: ABA and auxin-responsive, CGCGBOX: Multiple signal transduction, CURE: Cu and oxygen responsive, DPBF: ABA, DRE: Dehydration-responsive elements, GT1GMSAM4: Salt and pathogenesis-related, LTRE: Low temperature and cold-responsive, MYB: responsive to drought and ABA, MYC: Response to drought, cold and ABA, POLLEN: pollen and anther development, TKST1: Guard cell-specific gene expression, WBOXNTERF3: Wound signal and WBOXATNPR1: Salicylic acid-responsive, ERE: Ethylene-responsive elements, GARE: Gibberellic acid-responsive elements, TCA: Salicylic acid-responsive, TGACG: Me-Jasmonic acid-responsive).
