# Supplementary material for: Genome-scale identification, classification, and tissue specific expression analysis of late embryogenesis abundant (LEA) genes under abiotic stress conditions in Sorghum bicolor L
Source: PLoS One. 2019 Jan 16;14(1):e0209980. doi: 10.1371/journal.pone.0209980 (PMC6335061; doi:10.1371/journal.pone.0209980)
Supplement: S5 Table — (DOCX) [file pone.0209980.s008.docx]

| Sorghum  **S5 Table.** Calculations of synonymous and non synonymous substitutions rates of *SbLEA* orthologous genes in *Oryza* and *Arabidopsis* | Family | Chr | Ortholog | Family | Chr | **No. of Synonymous sites (S)** | **No. of Non -synonymous sites (N)** | **Non -synonymous substitution rate (d_N_)** | **Synonymous substitution rate (d_S_)** | d_N_ / d_S_ |
| --- | --- | --- | --- | --- | --- | --- | --- | --- | --- | --- |
| Sb01g002130 | SbLEA2-3 | 1 | At2g46140 | AtLEA-2 | 2 | 103.0 | 395.0 | 7.6694 | 0.0775 | 99.0000 |
| Sb01g036790 | SbLEA4-1 | 1 | At3g53040 | AtLEA-4 | 3 | 199.4 | 856.4 | 15.9914 | 0.1615 | 99.00 |
| Sb01g030000 | SbLEA2-7 | 1 | At4g39130 | AtDHN | 4 | 101.0 | 352.0 | 2.2339 | 2.2103 | 1.0107 |
| Sb01g046000 | SbLEA4-2 | 1 | At1g72100 | AtLEA-4 | 1 | 251.3 | 801.7 | 7.8820 | 9.6426 | 0.8174 |
| Sb02g030840 | SbLEA2-14 | 2 | At1g03120 | AtSMP | 1 | 99.4 | 446.6 | 5.1921 | 0.3990 | 13.0122 |
| Sb02g028010 | SbLEA6-1 | 2 | At2g41260 | AtATM | 2 | 71.9 | 279.1 | 16.3076 | 0.1647 | 99.0 |
| Sb03g012950 | SbLEA3-4 | 3 | At3g50980 | AtDHN | 3 | 53 | 181.0 | 16.7570 | 0.1693 | 99.0000 |
| Sb04g032400 | SbLEA2-25 | 4 | At1g76180 | AtDHN | 1 | 99.4 | 446.6 | 5.1921 | 0.3990 | 13.0122 |
| Sb04g023155 | SbLEA2-23 | 4 | At4g15910 | AtLEA-3 | 4 | 58.4 | 253.6 | 14.6777 | 5.7191 | 2.5664 |
| Sb09g018000 | SbLEA3-7 | 9 | At1g01470 | AtLEA-2 | 1 | 51.1 | 185.9 | 7.7729 | 0.0785 | 99.0000 |
| Sb09g027110 | SbLEA4-5 | 9 | At3g02480 | AtLEA-4 | 3 | 57.0 | 147.0 | 2.0026 | 41.3348 | 0.0484 |
| Sb10g003700 | SbDHN-6 | 10 | At5g66400 | AtDHN | 5 | 134.2 | 420.8 | 3.0740 | 2.3746 | 1.2945 |
| Sb01g046490 | SbSMP-2 | 1 | Os03g06360 | OsSMP | 3 | 202.8 | 625.2 | 2.1849 | 1.2118 | 1.8031 |
| Sb01g008210 | SbSMP-1 | 1 | Os03g07180 | OsLEA-4 | 3 | 173.6 | 630.4 | 6.3842 | 0.0645 | 99.0000 |
| Sb02g006180 | SbLEA2-9 | 2 | Os03g53610 | OsSMP | 3 | 158.7 | 474.3 | 17.2921 | 0.1747 | 99.0000 |
| Sb02g018540 | SbLEA1-1 | 2 | Os03g20680 | OsLEA-4 | 3 | 241.8 | 790.2 | 8.1134 | 0.0820 | 99.0000 |
| Sb03g033900 | SbLEA2-20 | 3 | Os01g50700 | OsDHN | 1 | 145.1 | 625.9 | 9.8897 | 0.0999 | 99.0000 |
| Sb03g012940 | SbLEA3-3 | 3 | Os01g21250 | OsLEA-3 | 1 | 64.9 | 214.1 | 1.1888 | 1.1757 | 1.0111 |
| Sb04g009840 | SbLEA2-21 | 4 | Os03g62620 | OsLEA-2 | 3 | 147.8 | 608.2 | 7.8123 | 34.3545 | 0.2274 |
| Sb04g032250 | SbLEA2-24 | 4 | Os02g44870 | OsDHN | 2 | 182.0 | 667.0 | 1.9310 | 1.1419 | 1.6910 |
| Sb06g028110 | SbLEA4-4 | 6 | Os04g52110 | OsLEA-4 | 4 | 190.5 | 631.5 | 7.9504 | 0.0803 | 99.0000 |
| Sb06g026900 | SbLEA1-1 | 6 | Os04g49980 | OsLEA-1 | 4 | 84.0 | 225.0 | 0.5923 | 0.2975 | 1.9908 |
| Sb07g007690 | SbLEA1-2 | 7 | Os08g23870 | OsLEA-1 | 8 | 99.3 | 350.7 | 0.6647 | 0.5149 | 1.2908 |
| Sb07g022150 | SbLEA3-6 | 7 | Os08g34990 | OsLEA-3 | 8 | 71.8 | 234.2 | 1.3879 | 50.8833 | 0.0273 |
| Sb09g018420 | SbDHN-4 | 9 | Os11g26790 | OsDHN | 11 | 82.5 | 373.5 | 4.3669 | 0.2051 | 21.2957 |
| Os02g35650 | OsLEA-3 | 2 | At4g02380 | AtLEA-3 | 4 | 62.5 | 210.5 | 7.4885 | 0.0756 | 99.0000 |
| Os03g45280 | OsDHN | 3 | At1g54410 | ATDHN | 1 | 58.8 | 217.2 | 1.3603 | 2.1942 | 0.6200 |
| Os03g53620 | OsSMP | 3 | At2g41280 | ATATM | 2 | 71.8 | 249.2 | 13.8298 | 0.1397 | 99.0000 |
| Os05g28210 | OsLEA-5 | 5 | At3g51810 | AtLEA-5 | 3 | 54.1 | 230.9 | 16.0058 | 0.1617 | 99.0000 |
| Os06g21910 | OsLEA-1 | 6 | At5g06760 | AtLEA-1 | 5 | 54.8 | 188.2 | 1.1328 | 0.4567 | 2.4804 |
| Os11g26750 | OsDHN | 11 | At2g23110 | Similar to LEA | 2 | 48.2 | 227.8 | 4.6370 | 0.0468 | 99.0000 |

(**d_N_ / d_S_ >1 = Positive or Darwinian Selection (Driving Change); d_N_ / d_S_ <1 = Purifying or Stabilizing Selection (Acting against change);**

**d_N_ / d_S_ =1 Neutral Selection** )
