# Supplementary material for: Genome-scale identification, classification, and tissue specific expression analysis of late embryogenesis abundant (LEA) genes under abiotic stress conditions in Sorghum bicolor L
Source: PLoS One. 2019 Jan 16;14(1):e0209980. doi: 10.1371/journal.pone.0209980 (PMC6335061; doi:10.1371/journal.pone.0209980)
Supplement: S6 Table — (DOCX) [file pone.0209980.s009.docx]

| S. No.  **S6 Table.** d_N_ / d_S_ ratios of SbLEA orthologs between *Sorghum, Setaria, Oryza, Brachypodium* and *Hordeum* | SbLEA | Ortholog | Score | Protein id | **Non -synonymous substitution rate (d_N_)** | **Synonymous substitution rate**  **(d_S_)** | **d_N_ / d_S_** |
| --- | --- | --- | --- | --- | --- | --- | --- |
| 1 | Sb02g018540 | *Setaria italica* | 98.5969 | K3ZXH5 | 8.6271 | 2.6824 | 3.2162 |
|  |  | *Hordeum vulgare* | 70.4774 | MOYE50 | 16.8170 | 0.1699 | 99.000 |
| 2 | Sb06g026900 | *Setaria italica* | 67.0106 | K3YBX7 | 0.4151 | 0.2893 | 1.4349 |
|  |  | *B. distachyon* | 54.299 | I1J150 | 0.4357 | 0.2840 | 1.5341 |
| 3 | Sb07g007690 | *Setaria italica* | 99.7525 | K3YK10 | 0.6215 | 0.5052 | 1.2303 |
|  |  | *B. distachyon* | 72.7886 | 1LHL22 | 16.7020 | 0.1687 | 99.0000 |
|  |  | *Oryza sativa* | 70.0922 | Q6ZOIO | 0.6647 | 0.5149 | 1.2908 |
| 4 | Sb10g000930 | *Setaria italica* | 63.929 | K3XZG5 | 16.0223 | 0.1618 | 99.0000 |
|  |  | *Oryza sativa* | 62.003 | Q8H683 | 15.7626 | 0.1592 | 99.0000 |
|  |  | *B. distachyon* | 60.8474 | I1H1W8 | 15.7705 | 0.1593 | 99.0000 |
|  |  | *Hordeum vulgare* | 58.5362 | C9ELM9 | 14.4637 | 6.6587 | 2.1721 |
| 5 | Sb10g012265 | *N A* | NA | NA | NA | NA | NA |
| 6 | Sb01g000200 | *B. distachyon* | 241.891 | I1HP22 | 3.8699 | 5.7961 | 0.6677 |
|  |  | *Hordeum vulgare* | 118.627 | MOWRD6 | 9.3568 | 0.0945 | 99.0000 |
|  |  | *Oryza sativa* | 107.457 | Q8GVN7 | 3.7781 | 1.6774 | 2.2523 |
| 7 | Sb01g001660 | *Setaria italica* | 556.599 | K4ABE8 | 15.6804 | 0.1584 | 99.0000 |
|  |  | *Oryza sativa* | 553.132 | Q75LD9 | 0.1054 | 0.0947 | 1.1136 |
|  |  | *Hordeum vulgare* | 520.776 | MOUP66 | 7.8060 | 0.0788 | 99.0000 |
|  |  | *B. distachyon* | 518.85 | I1GL04 | 6.2594 | 0.0632 | 99.0000 |
| 8 | Sb01g002130 | *Setaria italica* | 309.301 | K4ALRO | 7.4717 | 0.0755 | 99.0000 |
|  |  | *Oryza sativa* | 283.878 | Q75LJ9 | 1.9607 | 51.3508 | 0.0382 |
|  |  | *B. distachyon* | 243.432 | I1GL57 | 16.5862 | 0.1675 | 99.0000 |
|  |  | *Hordeum vulgare* | 221.09 | MOWNM1 | 6.9568 | 0.0703 | 99.0000 |
| 9 | Sb01g011230 | *B. distachyon* | 293.508 | I1GPC5 | 12.2794 | 0.1240 | 99.0000 |
|  |  | *Oryza sativa* | 285.419 | Q10EQ8 | 6.9166 | 9.0058 | 0.7680 |
|  |  | *Hordeum vulgare* | 189.889 | M0ZAF8 | 9.6625 | 1.2254 | 7.8850 |
| 10 | Sb01g016860 | *Setaria italica* | 266.544 | K4AJY3 | 9.4150 | 25.2639 | 0.3727 |
|  |  | *B. distachyon* | 204.912 | I1GQQ6 | 4.5452 | 0.7619 | 5.9653 |
|  |  | *Oryza sativa* | 198.749 | Q5Z9B4 | 11.7402 | 0.1186 | 99.0000 |
|  |  | *Hordeum vulgare* | 194.897 | M0Z0Z7 | 14.8992 | 6.6770 | 2.2314 |
| 11 | Sb01g018550 | *Setaria italica* | 286.574 | K4ADN8 | 5.8396 | 8.1575 | 0.7159 |
|  |  | *B. distachyon* | 244.973 | I1I4Z2 | 6.5964 | 11.2102 | 0.5884 |
|  |  | *Hordeum vulgare* | 242.662 | MOXX93 | 9.1814 | 7.3254 | 1.2534 |
| 12 | Sb01g030000 | *Setaria italica* | 275.789 | K4AF37 | 10.0385 | 23.1511 | 0.4336 |
|  |  | *B. distachyon* | 215.698 | I1I5Y1 | 16.9790 | 0.1715 | 99.0000 |
|  |  | *Hordeum vulgare* | 204.912 | F2CTV5 | 0.9768 | 3.5979 | 0.2715 |
|  |  | *Oryza sativa* | 178.718 | Q336V2 | 15.5945 | 4.0509 | 3.8497 |
| 13 | Sb01g040310 | *Setaria italica* | 306.605 | K4AEH1 | 1.6522 | 54.0989 | 0.0305 |
|  |  | *Oryza sativa* | 298.516 | Q0DT94 | 5.1036 | 9.0659 | 0.5629 |
|  |  | *B. distachyon* | 280.026 | I1H777 | 1.8544 | 54.4678 | 0.0340 |
|  |  | *Hordeum vulgare* | 275.018 | M0YZ15 | 1.8680 | 11.4368 | 0.1633 |
| 14 | Sb02g006180 | *Setaria italica* | 823.543 | K3ZQI8 | 8.2964 | 0.0838 | 99.0000 |
|  |  | *Oryza sativa* | 775.007 | Q6ZIW9 | 8.8498 | 0.0894 | 99.0000 |
|  |  | *Hordeum vulgare* | 697.967 | MOZ8T6 | 10.1103 | 0.1021 | 99.0000 |
|  |  | *B. distachyon* | 600.512 | I1H2Q2 | 6.1592 | 11.4862 | 0.5362 |
| 15 | Sb02g008820 | *Setaria italica* | 543.117 | K4A201 | 2.2634 | 1.1922 | 1.8986 |
|  |  | *Oryza sativa* | 513.457 | Q6Z4J9 | 3.9124 | 5.1281 | 0.7629 |
|  |  | *B. distachyon* | 496.893 | I1H2A5 | 6.3766 | 0.0644 | 99.0000 |
|  |  | *Hordeum vulgare* | 3977.897 | MOUP66 | 17.9698 | 2.0475 | 8.7763 |
| 16 | Sb02g002730 | *Setaria italica* | 207.608 | K4ADN8 | 8.9721 | 0.0906 | 99.0000 |
|  |  | *B. distachyon* | 184.496 | I1I4Z2 | 16.6285 | 0.1680 | 99.0000 |
|  |  | *Hordeum vulgare* | 177.948 | MOXX93 | 3.9740 | 7.3071 | 0.5439 |
| 17 | Sb02g017900 | *Setaria italica* | 330.487 | K4A043 | 0.0464 | 0.0113 | 4.1186 |
|  |  | *B. distachyon* | 293.508 | I1IIZ3 | 0.1168 | 0.1423 | 0.8207 |
|  |  | *Oryza sativa* | 277.33 | Q6H4U3 | 3.1392 | 0.0317 | 99.0000 |
|  |  | *Hordeum vulgare* | 270.781 | F2EEA1 | 1.5757 | 1.4174 | 1.1117 |
| 18 | Sb02g025570 | *Setaria italica* | 194.512 | K4A2N7 | 4.4903 | 45.7035 | 0.0982 |
|  |  | *Hordeum vulgare* | 169.474 | MOUZB8 | 16.2026 | 0.1637 | 99.0000 |
|  |  | *Oryza sativa* | 166.007 | Q69PF4 | 8.1705 | 0.0825 | 99.0000 |
|  |  | *B. distachyon* | 162.155 | I1IQD1 | 16.1053 | 0.1627 | 99.0000 |
| 19 | Sb02g030840 | *Setaria italica* | 186.037 | K4A2P8 | 8.1155 | 0.0820 | 99.0000 |
|  |  | *Oryza sativa* | 184.496 | Q652J9 | 5.4125 | 10.1791 | 0.5317 |
|  |  | *B. distachyon* | 141.739 | I1IS55 | 4.5416 | 9.2654 | 0.4902 |
|  |  | *Hordeum vulgare* | 128.257 | MOVLT6 | 16.4542 | 0.1662 | 99.0000 |
| 20 | Sb02g035010 | *Setaria italica* | 222.246 | K4A3A7 | 3.9895 | 2.4003 | 1.6621 |
|  |  | *B. distachyon* | 173.326 | I1GTV4 | 16.4378 | 1.5948 | 10.3069 |
|  |  | *Hordeum vulgare* | 118.627 | MOXK31 | 10.2996 | 0.1040 | 99.0000 |
|  |  | *Oryza sativa* | 114.775 | Q69SA8 | 2.6520 | 5.1434 | 0.5156 |
| 21 | Sb02g035250 | *Hordeum vulgare* | 289.656 | MOWRD6 | 3.5710 | 21.5825 | 0.1655 |
|  |  | *B. distachyon* | 277.715 | I1GTT5 | 1.4297 | 2.5507 | 0.5605 |
|  |  | *Oryza sativa* | 276.944 | Q8GVN7 | 2.3042 | 15.0717 | 0.1529 |
| 22 | Sb02g038356 | *Setaria italica* | 208.764 | K4ADN8 | 16.5296 | 0.1670 | 99.0000 |
|  |  | *B. distachyon* | 187.193 | I1I4Z2 | 16.5983 | 0.1677 | 99.0000 |
|  |  | *Hordeum vulgare* | 181.03 | MOXX93 | 5.0972 | 2.6299 | 1.9382 |
| 23 | Sb03g001170 | *Setaria italica* | 266.544 | K3XMWO | 0.5854 | 0.4890 | 1.1972 |
|  |  | *B. distachyon* | 258.455 | I1HDG2 | 0.1549 | 0.0954 | 1.6236 |
|  |  | *Oryza sativa* | 256.529 | Q9AWZ5 | 0.6795 | 0.5942 | 1.1436 |
|  |  | *Hordeum vulgare* | 244.973 | MOWI75 | 0.6785 | 0.6465 | 1.0495 |
| 24 | Sb03g025840 | *B. distachyon* | 204.912 | I1HS8 | 8.4766 | 0.0856 | 99.0000 |
|  |  | *Oryza sativa* | 197.593 | Q8LQJ5 | 6.3377 | 4.8185 | 1.3153 |
|  |  | *Hordeum vulgare* | 176.792 | MOV5V5 | 15.1826 | 5.1572 | 2.9440 |
| 25 | Sb03g033900 | *Setaria italica* | 363.229 | K3XL97 | 2.6798 | 2.4286 | 1.1034 |
|  |  | *Oryza sativa* | 327.405 | Q942B1 | 8.1295 | 0.0821 | 99.0000 |
|  |  | *Hordeum vulgare* | 298.13 | MOWW20 | 16.3404 | 0.1651 | 99.0000 |
|  |  | *B. distachyon* | 294.278 | I1HRA6 | 2.1967 | 54.2902 | 0.0405 |
| 26 | Sb04g009840 | *Setaria italica* | 205.682 | K3YYU9 | 15.8807 | 0.1604 | 99.0000 |
|  |  | *B. distachyon* | 68.5514 | I1HHD3 | 15.1422 | 0.1530 | 99.0000 |
|  |  | *Hordeum vulgare* | 67.781 | MOWW20 | 12.5572 | 0.1268 | 99.0000 |
|  |  | *Oryza sativa* | 65.855 | Q65X87 | 13.6076 | 10.7491 | 1.2659 |
| 27 | Sb04g022010 | *Setaria italica* | 316.235 | K3YVP5 | 3.9144 | 5.7436 | 0.6815 |
|  |  | *Oryza sativa* | 268.855 | Q6ER87 | 4.3951 | 1.0837 | 4.0555 |
|  |  | *Hordeum vulgare* | 264.233 | MOVIPO | 3.9757 | 2.0350 | 1.9537 |
|  |  | *B. distachyon* | 263.077 | I1IAA7 | 16.3196 | 0.1648 | 99.0000 |
| 28 | Sb04g023155 | *Setaria italica* | 568.155 | K3YR48 | 8.4262 | 0.0851 | 99.0000 |
|  |  | *Oryza sativa* | 368.622 | QOEOC2 | 8.4521 | 0.0854 | 99.0000 |
|  |  | *B. distachyon* | 278.1 | I1IM72 | 3.6425 | 4.2233 | 0.8625 |
| 29 | Sb04g032250 | *Setaria italica* | 88.5817 | K3YUM9 | 1.1592 | 0.8054 | 1.4393 |
|  |  | *B. distachyon* | 79.337 | I1IYO7 | 2.4864 | 1.9751 | 1.2589 |
|  |  | *Oryza sativa* | 78.1814 | Q6ESR4 | 1.9310 | 1.1419 | 1.6910 |
|  |  | *Hordeum vulgare* | 75.8702 | MOUW32 | 3.3547 | 1.6968 | 1.9770 |
| 30 | Sb04g032400 | *Setaria italica* | 278.87 | K3YVW6 | 0.4406 | 0.3808 | 1.1571 |
|  |  | *Hordeum vulgare* | 197.978 | F2E0S7 | 4.7122 | 10.9346 | 0.4309 |
|  |  | *Oryza sativa* | 187.963 | Q6ESG7 | 4.5796 | 2.4442 | 1.8737 |
|  |  | *B. distachyon* | 135.576 | I1ICH5 | 7.4118 | 0.0749 | 99.0000 |
| 31 | Sb05g001340 | *Setaria italica* | 257.684 | K3Y9V7 | 0.2281 | 0.2721 | 0.8382 |
|  |  | *Hordeum vulgare* | 245.743 | M0V6X9 | 4.7246 | 41.1679 | 0.1148 |
|  |  | *Oryza sativa* | 234.955 | C7J9A0 | 16.7669 | 0.1694 | 99.0000 |
|  |  | *B. distachyon* | 231.876 | I1IUZ0 | 12.7515 | 0.1288 | 99.0000 |
| 32 | Sb05g003630 | *Hordeum vulgare* | 244.276 | M0YT51 | 10.4808 | 8.0603 | 1.3003 |
|  |  | *Oryza sativa* | 240.736 | Q2RB10 | 5.4112 | 11.3286 | 0.4777 |
|  |  | *B. distachyon* | 181.415 | I1I6T0 | 14.6333 | 7.7168 | 1.8963 |
|  |  | *Setaria italica* | 126.331 | K3XKE5 | 13.4414 | 0.1358 | 99.0000 |
| 33 | Sb05g003631 | *Setaria italica* | 327.02 | K3ZJV0 | 0.2004 | 0.1753 | 1.1433 |
|  |  | *Oryza sativa* | 315.849 | Q53NC9 | 1.0856 | 1.1103 | 0.9778 |
|  |  | *B. distachyon* | 311.997 | I1INE7 | 12.6426 | 14.1584 | 0.8929 |
|  |  | *Hordeum vulgare* | 308.145 | M0XD75 | 1.1455 | 1.7135 | 0.6685 |
| 34 | Sb06g016230 | *Setaria italica* | 369.392 | K3Y9Y2 | 0.1682 | 0.1626 | 1.0345 |
|  |  | *Oryza sativa* | 351.673 | Q7X7T9 | 0.2048 | 0.1983 | 1.0328 |
|  |  | *Hordeum vulgare* | 345.895 | F2EBE7 | 0.1958 | 0.1820 | 1.0759 |
|  |  | *B. distachyon* | 330.102 | I1IXL3 | 1.8247 | 1.2846 | 1.4204 |
| 35 | Sb06g029380 | *Setaria italica* | 347.821 | K3YCD5 | 2.7111 | 20.5617 | 0.1318 |
|  |  | *B. distachyon* | 266.159 | I1J251 | 1.1793 | 3.0101 | 0.3918 |
|  |  | *Oryza sativa* | 257.684 | Q7XN75 | 17.1430 | 0.1732 | 99.0000 |
|  |  | *Hordeum vulgare* | 101.293 | M0WRD6 | 3.2122 | 4.3247 | 0.7428 |
| 36 | Sb06g032920 | *Setaria italica* | 248.825 | K3ZC72 | 2.3424 | 1.7173 | 1.3640 |
|  |  | *Oryza sativa* | 232.261 | Q7XKB7 | 2.5108 | 4.4963 | 0.5584 |
|  |  | *Hordeum vulgare* | 231.876 | F2DX93 | 7.5274 | 0.0760 | 99.0000 |
|  |  | *B. distachyon* | 216.468 | I1J3C8 | 1.7702 | 1.4353 | 1.2333 |
| 37 | Sb06g033570 | *Oryza sativa* | 255.373 | Q7XPU1 | 0.2228 | 0.3334 | 0.6682 |
|  |  | *B. distachyon* | 249.21 | I1J3H9 | 0.2642 | 0.3319 | 0.7958 |
|  |  | *Setaria italica* | 218.779 | K3Z9P4 | 15.5507 | 4.5073 | 3.4501 |
| 38 | Sb06g033580 | *Setaria italica* | 305.449 | K3Z7Z1 | 2.1479 | 50.4837 | 0.0425 |
|  |  | *Hordeum vulgare* | 298.13 | M0V0K6 | 3.8839 | 0.7548 | 5.1456 |
|  |  | *Oryza sativa* | 293.123 | Q7XPU0 | 8.0367 | 0.0812 | 99.0000 |
|  |  | *B. distachyon* | 281.182 | I1J3H8 | 3.2212 | 1.0324 | 3.1202 |
| 39 | Sb07g000360 | *Setaria italica* | 287.345 | K3YKZ6 | 2.8083 | 2.2635 | 1.2407 |
|  |  | *B. distachyon* | 221.09 | I1I095 | 12.2635 | 0.1239 | 99.0000 |
|  |  | *Oryza sativa* | 152.91 | Q7XPU1 | 8.4556 | 0.0854 | 99.0000 |
| 40 | Sb08g001610 | *Oryza sativa* | 230.335 | Q2QY99 | 10.8065 | 21.3016 | 0.5073 |
|  |  | *Hordeum vulgare* | 223.016 | M0YT51 | 8.2101 | 30.8834 | 0.2658 |
|  |  | *B. distachyon* | 169.088 | I1I6T0 | 12.3937 | 15.1413 | 0.8185 |
|  |  | *Setaria italica* | 122.479 | K3XKE5 | 6.0718 | 0.3961 | 15.3274 |
| 41 | Sb08g003690 | *Setaria italica* | 251.906 | K3YA32 | 9.5550 | 0.0965 | 99.0000 |
|  |  | *Oryza sativa* | 169.859 | Q2QXF5 | 10.5894 | 0.1070 | 99.0000 |
|  |  | *Hordeum vulgare* | 166.777 | F2D8F9 | 15.7781 | 4.4733 | 3.5272 |
|  |  | *B. distachyon* | 161.77 | I1IU72 | 1.8861 | 1.3544 | 1.3926 |
| 42 | Sb08g003720 | *Setaria italica* | 308.531 | K3YA19 | 14.0975 | 9.6623 | 1.4590 |
|  |  | *Hordeum vulgare* | 305.834 | M0YCK9 | 16.0736 | 0.1624 | 99.0000 |
|  |  | *Oryza sativa* | 302.753 | Q2QXF1 | 16.1289 | 0.1629 | 99.0000 |
|  |  | *B. distachyon* | 302.368 | I1IU68 | 14.5302 | 0.1468 | 99.0000 |
| 43 | Sb09g023690 | *Setaria italica* | 271.166 | K3Z9K0 | 3.6583 | 1.2344 | 2.9636 |
|  |  | *Hordeum vulgare* | 200.675 | M0VPY8 | 14.3288 | 8.5817 | 1.6697 |
|  |  | *B. distachyon* | 182.185 | I1HIB9 | 5.2079 | 15.1356 | 0.3441 |
|  |  | *Oryza sativa* | 120.939 | Q0IQI1 | 5.2967 | 38.3217 | 0.1382 |
| 44 | Sb09g026230 | *Setaria italica* | 258.84 | K3Z8Y2 | 6.1566 | 0.0622 | 99.0000 |
|  |  | *Oryza sativa* | 203.371 | Q65X87 | 16.7878 | 0.1696 | 99.0000 |
|  |  | *B. distachyon* | 180.259 | I1HHD3 | 1.9354 | 49.5945 | 0.0390 |
|  |  | *Hordeum vulgare* | 159.458 | M0VUR0 | 2.6971 | 15.6648 | 0.1722 |
| 45 | Sb09g029870 | *Setaria italica* | 162.155 | K3ZFH5 | 8.3985 | 16.3825 | 0.5126 |
|  |  | *Hordeum vulgare* | 148.288 | M0WZP2 | 0.9611 | 48.8868 | 0.0197 |
|  |  | *Oryza sativa* | 144.05 | Q75HZ1 | 6.7041 | 18.8351 | 0.3559 |
|  |  | *B. distachyon* | 132.88 | I1HG23 | 11.3289 | 19.3592 | 0.5852 |
| 46 | Sb01g033070 | *Setaria italica* | 70.0992 | K4AGJ2 | 15.2577 | 0.1541 | 99.0000 |
|  |  | *B. distachyon* | 63.1586 | I1H4R3 | 12.9234 | 13.2498 | 0.9754 |
|  |  | *Oryza sativa* | 59.3066 | B9F0L9 | 6.0052 | 0.2945 | 20.3938 |
| 47 | Sb03g009860 | *Setaria italica* | 53.9138 | K3XPD4 | 15.0988 | 6.8546 | 2.2027 |
|  |  | *Oryza sativa* | 50.0618 | Q1EHU2 | 16.5904 | 1.0821 | 15.3323 |
| 48 | Sb03g012940 | *Setaria italica* | 103.605 | K3XNL8 | 10.6855 | 20.7931 | 0.5139 |
|  |  | *Oryza sativa* | 95.9005 | Q0JN91 | 1.1888 | 1.1757 | 1.0111 |
|  |  | *Hordeum vulgare* | 82.8037 | F2DB42 | 1.0875 | 0.9594 | 1.1336 |
|  |  | *B. distachyon* | 77.0258 | I1HEY8 | 1.9445 | 2.7392 | 0.7099 |
| 49 | Sb03g012950 | *Setaria italica* | 70.4774 | K3XNM4 | 16.8745 | 0.1704 | 99.0000 |
|  |  | *Oryza sativa* | 58.151 | Q0JN91 | 6.9220 | 3.1595 | 2.1909 |
|  |  | *Hordeum vulgare* | 55.0694 | F2DB42 | 14.9960 | 6.9157 | 2.1684 |
| 50 | Sb04g023310 | *-NA--* | NA | NA | NA | NA | NA |
| 51 | Sb07g022150 | *Setaria italica* | 75.0998 | K3YKZ1 | 9.3724 | 0.0947 | 99.0000 |
|  |  | *Oryza sativa* | 62.3882 | Q6ZLD9 | 17.0135 | 0.2319 | 73.3722 |
|  |  | *B. distachyon* | 52.7582 | I1IAN8 | 16.8439 | 0.1701 | 99.0000 |
| 52 | Sb09g018000 | *Setaria italica* | 137.502 | K3ZDW6 | 6.7791 | 4.8073 | 1.4102 |
|  |  | *Oryza sativa* | 129.798 | Q60E81 | 7.9075 | 0.0799 | 99.0000 |
|  |  | *B. distachyon* | 108.612 | I1HK78 | 15.3002 | 0.1545 | 99.0000 |
| 53 | Sb01g036790 | *Hordeum vulgare* | 340.502 | F2ECH4 | 3.8096 | 0.0385 | 99.0000 |
|  |  | *Setaria italica* | 335.109 | K4AJK1 | 16.6826 | 0.1685 | 99.0000 |
|  |  | *B. distachyon* | 335.109 | I1H5Z6 | 7.3798 | 0.0745 | 99.0000 |
|  |  | *Oryza sativa* | 321.627 | A3AHG5 | 6.1451 | 0.0621 | 99.0000 |
| 54 | Sb01g046000 | *Setaria italica* | 152.14 | K4ABN0 | 5.8312 | 0.0589 | 99.0000 |
|  |  | *Oryza sativa* | 114.775 | Q8S7U3 | 3.0446 | 2.9493 | 1.0323 |
|  |  | *B. distachyon* | 87.8113 | I1H9A9 | 15.9685 | 7.7837 | 2.0515 |
| 55 | Sb03g032380 | *Setaria italica* | 206.838 | K3XLP0 | 17.7789 | 1.1238 | 15.8202 |
|  |  | *Oryza sativa* | 133.65 | Q94JF2 | 8.4882 | 5.1677 | 1.6425 |
|  |  | *B. distachyon* | 132.109 | I1HQS3 | 16.3834 | 4.8223 | 3.3974 |
|  |  | *Hordeum vulgare* | 111.309 | F2CRD9 | 17.5944 | 1.9806 | 8.8834 |
| 56 | Sb06g028110 | *Setaria italica* | 351.673 | K3Y8Z0 | 14.1846 | 9.0387 | 1.5693 |
|  |  | *Oryza sativa* | 214.542 | Q7XPL4 | 7.9504 | 0.0803 | 99.0000 |
|  |  | *B. distachyon* | 211.075 | I1J1M7 | 6.9201 | 0.0699 | 99.0000 |
|  |  | *Hordeum vulgare* | 171.785 | M0UUA6 | 6.5489 | 0.0662 | 99.0000 |
| 57 | Sb09g027110 | *Setaria italica* | 67.3958 | K3Z9P0 | 15.8855 | 0.1605 | 99.0000 |
|  |  | *B. distachyon* | 56.9954 | I1HGZ9 | 15.9002 | 0.1606 | 99.0000 |
| 58 | Sb09g016830 | *Setaria italica* | 217.238 | K3ZAF1 | 15.3896 | 2.6173 | 5.8801 |
|  |  | *B. distachyon* | 138.272 | I1HKD9 | 2.9432 | 57.9853 | 0.0508 |
|  |  | *Oryza sativa* | 107.457 | P46520 | 4.3435 | 54.5540 | 0.0796 |
| 59 | Sb02g028010 | *Setaria italica* | 141.739 | K4A2X5 | 0.2783 | 0.3854 | 0.7220 |
|  |  | *B. distachyon* | 118.627 | I1IR57 | 1.6102 | 0.8629 | 1.8661 |
|  |  | *Hordeum vulgare* | 109.383 | M0YLU3 | 1.8348 | 1.3789 | 1.3307 |
| 60 | Sb01g008210 | *Setaria italica* | 366.696 | K4AIE4 | 0.7252 | 0.7584 | 0.9563 |
|  |  | *B. distachyon* | 325.094 | I1GNC1 | 16.7469 | 0.1692 | 99.0000 |
|  |  | *Oryza sativa* | 284.263 | Q0DNL6 | 9.7117 | 0.0981 | 99.0000 |
| 61 | Sb01g046490 | *Setaria italica* | 323.168 | K4AJX5 | 1.5545 | 1.5116 | 1.0284 |
|  |  | *Oryza sativa* | 245.358 | Q8H8B0 | 2.1849 | 1.2118 | 1.8031 |
|  |  | *B. distachyon* | 237.369 | I1H9I4 | 16.9613 | 0.1713 | 99.0000 |
|  |  | *Hordeum vulgare* | 218.009 | M0Z5Y1 | 17.2702 | 0.1744 | 99.0000 |
| 62 | Sb07g015410 | *Setaria italica* | 211.46 | K3YJU5 | 16.6316 | 0.1680 | 99.0000 |
|  |  | *Oryza sativa* | 159.458 | Q5Z9Y8 | 10.0442 | 0.1015 | 99.0000 |
|  |  | *Hordeum vulgare* | 146.747 | F2DNE8 | 16.3592 | 0.1652 | 99.0000 |
|  |  | *B. distachyon* | 127.102 | I1IIE4 | 16.4833 | 0.1665 | 99.0000 |
| 63 | Sb03g027020 | *Setaria italica* | 216.53 | K3XGD3 | 4.0336 | 1.0019 | 4.0259 |
|  |  | *B. distachyon* | 152.14 | I1HQR1 | 13.9684 | 10.5275 | 1.3268 |
|  |  | *Oryza sativa* | 144.05 | P30287 | 6.1634 | 0.0623 | 99.0000 |
|  |  | *Hordeum vulgare* | 97.4413 | M0V920 | 17.3149 | 2.2940 | 7.5479 |
| 64 | Sb03g032255 | *Setaria italica* | 164.851 | K3XGD3 | 5.2334 | 0.0529 | 99.0000 |
|  |  | *B. distachyon* | 115.546 | I1HQR1 | 1.5125 | 2.3111 | 0.6545 |
|  |  | *Oryza sativa* | 101.679 | P30287 | 16.5607 | 0.1673 | 99.0000 |
|  |  | *Hordeum vulgare* | 97.413 | M0V920 | 0.8091 | 0.8617 | 0.9390 |
| 65 | Sb03g037700 | *Setaria italica* | 322.013 | K3XKE5 | 0.9942 | 1.1939 | 0.8327 |
|  |  | *Oryza sativa* | 280.411 | Q8RUR5 | 10.1507 | 0.1025 | 99.0000 |
|  |  | *B. distachyon* | 252.292 | I1HSP6 | 8.1645 | 0.0825 | 99.0000 |
|  |  | *Hordeum vulgare* | 172.94 | M0WDM0 | 4.8307 | 2.9190 | 1.6549 |
| 66 | Sb09g018420 | *Setaria italica* | 72.4034 | K3ZK21 | 2.9194 | 0.1386 | 21.0571 |
|  |  | *B. distachyon* | 68.5514 | I1GXT7 | 15.8481 | 0.2632 | 60.2244 |
|  |  | *Oryza sativa* | 61.0106 | Q2R4Z4 | 4.3669 | 0.2051 | 21.2956 |
| 67 | Sb09g029860 | *Oryza sativa* | 256.914 | Q75HZ0 | 3.7384 | 6.2330 | 0.5998 |
|  |  | *B. distachyon* | 229.95 | I1HG22 | 3.0422 | 7.9453 | 0.3829 |
|  |  | *Hordeum vulgare* | 227.639 | M0Z9N7 | 7.3750 | 0.0745 | 99.0000 |
|  |  | *Setaria italica* | 213.386 | K3XPF0 | 9.1577 | 0.0925 | 99.0000 |
| 68 | Sb10g003700 | *Setaria italica* | 70.8626 | K3ZJ30 | 15.6793 | 4.4521 | 3.5218 |
|  |  | *B. distachyon* | 57.3806 | I1ILU4 | 2.4192 | 3.7143 | 0.6513 |
|  |  | *Oryza sativa* | 53.1434 | Q2R4Z5 | 3.4843 | 41.0801 | 0.0848 |

(**d_N_ / d_S_ >1 = Positive or Darwinian Selection (Driving Change); d_N_ / d_S_ <1 = Purifying or Stabilizing Selection**

**(Acting against change); d_N_ / d_S_ =1 Neutral Selection** )
