# Supplementary material for: Genome-scale identification, classification, and tissue specific expression analysis of late embryogenesis abundant (LEA) genes under abiotic stress conditions in Sorghum bicolor L
Source: PLoS One. 2019 Jan 16;14(1):e0209980. doi: 10.1371/journal.pone.0209980 (PMC6335061; doi:10.1371/journal.pone.0209980)
Supplement: S7 Table — (DOCX) [file pone.0209980.s010.docx]

| **Genes**  **S7 Table.** Native and relative **expression analysis of SbLEAs** | **Tissues and stresses** | | | | | | | | | | | | | | |
| --- | --- | --- | --- | --- | --- | --- | --- | --- | --- | --- | --- | --- | --- | --- | --- |
|  | **R** | **S** | **L** | **DR** | **SR** | **HR** | **CR** | **DS** | **SS** | **HS** | **CS** | **DL** | **SL** | **HL** | **CL** |
| **SbLEA1-2** | 0.462 | -0.290 | -0.172 | 1.9543 | 6.3642 | 14.22148 | 0.130308 | 0.45376 | 19.33755 | 0.199805 | 1.295342 | 5.95183 | 1.628263 | 0.360149 | 14.65512 |
| **SbLEA1-3** | 0.237 | -0.544 | 0.307 | 1.4012 | 2.1734 | 1.972465 | 0.065154 | 0.233258 | 2.302711 | 0.553504 | 0.404254 | 0.511687 | 0.854607 | 0.107818 | 2.485151 |
| **SbLEA1-5** | -0.234 | -0.702 | 0.935 | 11.287 | 4.9933 | 0.376312 | 0.239816 | 0.50698 | 0.430276 | 1.254112 | 15.06714 | 1.457336 | 5.004873 | 0.181327 | 2.163449 |
| **SbLEA2-2** | -0.672 | 0.261 | 0.410 | 0.6056 | 0.0105 | 0.156041 | 0.25349 | 0.420448 | 0.354371 | 1.823445 | 0.515246 | 0.571701 | 1.572798 | 0.047586 | 0.786399 |
| **SbLEA2-6** | 0.287 | -0.235 | -0.052 | 4.2183 | 1.5583 | 3.758091 | 0.790041 | 0.61132 | 7.799239 | 1.643381 | 38.67511 | 9.940603 | 0.135216 | 0.053909 | 16.03701 |
| **SbLEA2-9** | 0.207 | -0.396 | 0.189 | 5.1933 | 15.889 | 28.2465 | 0.408951 | 1.494849 | 42.32192 | 0.777366 | 3.845932 | 7.276947 | 7.37853 | 0.97716 | 23.97291 |
| **SbLEA2-13** | -0.017 | -0.402 | 0.419 | 0.3503 | 0.0620 | 0.057114 | 0.011518 | 0.005524 | 10.22007 | 5.302478 | 25.3398 | 20.01949 | 2.286805 | 0.39685 | 22.5231 |
| **SbLEA2-15** | -0.084 | -0.289 | 0.373 | 0.7992 | 0.1672 | 1.214195 | 0.510506 | 0.543367 | 1.561934 | 0.46544 | 11.65869 | 1.639588 | 0.240371 | 0.750886 | 1.794191 |
| **SbLEA2-18** | 0.347 | -0.970 | 0.623 | 0.1503 | 0.9395 | 0.381565 | 0.993092 | 6.634556 | 2.467984 | 1.271619 | 59.85224 | 3.348078 | 4.237852 | 2.065751 | 2.10429 |
| **SbLEA2-23** | -0.314 | 1.478 | 0.000 | 1.2804 | 0.0026 | 369.6459 | 8.111676 | 0.514057 | 7.585968 | 2.543238 | 0.174746 | 191.7833 | 6.789632 | 0.016139 | 340.9304 |
| **SbLEA2-37** | 0.198 | 0.005 | -0.203 | 2.3402 | 5.8158 | 111.4305 | 0.115023 | 0.283221 | 41.45095 | 0.449585 | 0.842842 | 48.95327 | 9.339422 | 0.049951 | 99.27326 |
| **SbLEA2-40** | 0.102 | 0.330 | -0.432 | 1.3915 | 0.3275 | 7.110741 | 0.000108 | 0.067452 | 10.14948 | 0.221698 | 0.454809 | 3.466148 | 1.030492 | 0.026583 | 6.696157 |
| **SbLEA3-2** | 0.645 | -0.389 | -0.255 | 0.7719 | 15.242 | 133.4356 | 0.933033 | 36.00187 | 425.5938 | 1.146047 | 13.1167 | 52.1044 | 82.32926 | 14.09065 | 434.5364 |
| **SbLEA3-4** | 0.313 | -0.287 | -0.025 | 0.1005 | 1.3286 | 1.536875 | 0.10083 | 0.795536 | 0.703847 | 2.037312 | 9.601989 | 1.963372 | 0.302149 | 0.06871 | 20.58231 |
| **SbLEA3-7** | -0.091 | -0.250 | 0.342 | 4.8121 | 4.6589 | 3.010493 | 0.070316 | 0.210224 | 2.894538 | 0.130007 | 0.922316 | 0.501157 | 1.233992 | 0.108568 | 1.896492 |
| **SbLEA4-1** | 0.444 | -0.816 | 0.372 | 0.0431 | 0.1036 | 0.016289 | 0.001797 | 4.723971 | 4.297011 | 0.686184 | 0.012035 | 4.479486 | 0.058046 | 0.02057 | 6.884411 |
| **SbLEA4-2** | 0.065 | -0.516 | 0.451 | 1.2454 | 1.5475 | 0.888843 | 0.262429 | 0.426317 | 1.127661 | 0.425334 | 2.854689 | 0.454809 | 0.948246 | 0.094514 | 1.437272 |
| **SbLEA4-3** | -0.058 | -0.602 | 0.660 | 0.4137 | 2.5847 | 0.225313 | 1 | 0.76313 | 1.628263 | 1.60956 | 39.48775 | 2.224272 | 2.004626 | 0.173941 | 3.256525 |
| **SbLEA4-4** | -0.271 | -0.408 | 0.679 | 1.2198 | 3.4581 | 0.993092 | 0.473029 | 0.469761 | 1.251218 | 1.298339 | 2.178497 | 0.382447 | 1.709214 | 0.106333 | 1.388313 |
| **SbLEA5-1** | -0.172 | -0.113 | 0.285 | 0.9308 | 0.0180 | 0.153893 | 0.216134 | 0.035649 | 0.474123 | 0.186425 | 0.595979 | 0.207809 | 0.021692 | 0.006115 | 0.600124 |
| **SbLEA6-1** | 0.139 | 0.000 | 1.037 | 0.7103 | 3.0737 | 0.11744 | 0.619854 | 0.133972 | 1.251218 | 0.011896 | 1.498307 | 0.012898 | 6.119159 | 0.107073 | 1.76949 |
| **SbSMP-1** | -0.228 | -0.036 | 0.264 | 0.8685 | 0.1830 | 3.944931 | 3.317278 | 1.156688 | 7.077959 | 6.573523 | 14.35353 | 6.558352 | 1.331759 | 0.048585 | 6.884411 |
| **SbSMP-2** | -0.027 | -0.491 | 0.518 | 16.990 | 10.852 | 5.9380 | 0.482968 | 0.066523 | 13.86459 | 0.290511 | 0.011706 | 6.789632 | 11.65869 | 0.943874 | 5.4014 |

(R; Root, S: Stem, L: Leaf, D: Drought, S: Salt, H: Heat, C: Cold)
